# Feasibility of the HEAR-aware App for Hearing Loss Self-Management: A Nonrandomized Intervention Study to Examine Intervention Acceptability and the Stages-of-Change Concept

**DOI:** 10.1097/AUD.0000000000001414

**Published:** 2023-09-06

**Authors:** Marieke Pronk, Vanessa Feenstra-Kikken, Cas Smits, Jana Besser, Birgit I. Lissenberg-Witte, Conny Polleunis, Barbra H.B. Timmer, Sophia E. Kramer

**Affiliations:** 1 Otolaryngology—Head and Neck Surgery, Section Ear and Hearing, Amsterdam UMC location Vrije Universiteit Amsterdam, Amsterdam Public Health Research Institute, Amsterdam, the Netherlands; 2 Sonova AG, Stäfa, Switzerland; 3 Epidemiology & Data Science, Amsterdam UMC location Vrije Universiteit Amsterdam, Amsterdam, the Netherlands; 4 Sonova Audiological Care Nederland BV (Schoonenberg HoorSupport), Rotterdam, the Netherlands; 5 School of Health and Rehabilitation Sciences, The University of Queensland, St. Lucia, Brisbane, Australia.

**Keywords:** Adults, Ecological Momentary Assessment, Ecological momentary intervention, E-health, Hearing loss, Intervention, Self-management, Smartphone app, Stages-of-change

## Abstract

**Objectives:**

The HEAR-aware project targets adults ≥50 years who were recently diagnosed with hearing loss and declined hearing aids, but were open for support via a smartphone app on different target behaviors (TBs). The HEAR-aware app, based on Ecological Momentary Assessment and Ecological Momentary Intervention (EMA, EMI), contains educational materials (“snippets”) tailored partly to the user’s experienced listening situations. The app aims to increase adults’ TB-specific readiness to take action on hearing problems. The present study focused on examining feasibility regarding three novel aspects: (1) the app’s acceptability, mainly regarding its EMA and EMI elements (compliance, usability, usefulness, satisfaction), (2) psychometric properties of 10 new TB-specific stages-of-change (SoC) measures (test–retest reliability, construct validity), and (3) the potential of tailoring snippets on a person’s SoC.

**Design:**

A nonrandomized intervention study including four measurements with 2-week intervals (T0–T3). (1) The intervention period lasted 4 weeks. App usage data were collected throughout (T1–T3). Usability, usefulness, and satisfaction were measured at T3 (n = 26). (2) Reliability concerned T0 and T1 data, in between which no intervention occurred. Intraclass correlation coefficients (ICCs) were calculated (n = 29). Construct validity was examined by calculating correlations between the different TB-specific scales (at T0), and also between each of them and self-reported hearing disability (n = 29). (3) Person-tailoring by SoC was examined using T0 and T1 data. Linear mixed models were applied to test whether users rated snippets corresponding to their SoC as more interesting and useful than noncorresponding snippets (n = 25).

**Results:**

(1) The percentage of participants that complied with the intended usage varied across the five predefined compliance criteria (lowest: 8%; highest: 85%). Median snippet satisfaction scores were reasonably positive (3.5 to 4.0 of 5). Usability was good (System Usability Score, mean = 72.4, SD = 14.3) and usefulness satisfactory (Intrinsic Motivation Inventory, mean = 4.4, SD = 1.4), but showed large variance. (2) The 10 TB-specific scales showed fair-to-excellent reliabilities (range ICCs = 0.51 to 0.80). Correlations between the TB-specific scales ranged between −0.17 (*p* > 0.05) and 0.74 (*p* < 0.001), supporting only partly overlap between their underlying constructs. Only the correlation between TB-specific readiness for hearing aid uptake and self-reported hearing disability was significant. (3) Correspondence of a snippet’s SoC with the person’s SoC significantly related to “interesting” ratings (*p* = 0.006). Unexpectedly, for snippets with a lower SoC than the participant’s, further deviation of the snippet’s SoC from the participant’s SoC, increased the participant’s interest in the snippet. The relationship with “usefulness” was borderline significant.

**Conclusions:**

(1) Overall usability, usefulness, and satisfaction scores indicated sufficient app acceptability. The high variance and fairly low compliance showed room for improving the app’s EMA/EMI parts for part of the participants. (2) The 10 new TB-specific SoC measures showed sufficient reliability, supporting that they measured different types of readiness to take action on hearing problems (construct validity). (3) The unexpected findings regarding tailoring educational app materials to individuals’ SoC deserve further study.

## INTRODUCTION

Hearing aid fitting is the most common form of rehabilitation for adults with hearing loss (HL), with beneficial outcomes on disability and wellbeing (e.g., Chisolm et al. 2004, 2007). Nonetheless, an alarming two-thirds of adults do not take up hearing aids (Hartley et al. 2010). Other forms of support are sparsely offered. Thus, there is a large group that stays unserved thereby contributing significantly to the HL public health disease burden (Orji et al. 2020).

Previous studies showed that self-management support programs (SMSPs) delivered via e-health (e-SMSPs) have the potential to reach many adults with valuable knowledge and skills needed to manage the impact of HL on their wellbeing (Kramer et al. 2005; Hickson et al. 2007, 2019; Thorén et al. 2014; Ferguson et al. 2016; Preminger & Rothpletz 2016; Meijerink et al. 2020). Effective ingredients of SMSPs include training of skills (e.g., communication strategies, emotional coping, hearing aid handling), facilitating social support, and transfer of knowledge (e.g., about HL etiology and consequences, hearing aid efficacy).

Present e-SMSPs however hold limitations. First, because most of them are not offered independently of hearing aids (e.g., Thorén et al. 2014; Ferguson et al. 2016; Meijerink et al. 2020), adults with HL without hearing aids are not reached. Second, many existing e-SMSPs offer comprehensive, modular, and time-consuming programs that should be followed in chronological order (e.g., Thorén et al. 2014; Hickson et al. 2019), limiting their acceptability and compliance to them (Laplante-Lévesque et al. 2010; Meijerink et al. 2020). To resolve this, content in small, accessible, stand-alone pieces may be needed (Pronk et al. 2020). Third, existing e-SMSPs do not include elements specifically designed to elicit awareness of hearing limitations. This seems important, as unawareness is considered a cause of passivity in hearing problems (Knudsen et al. 2010; Pronk et al. 2017; Timmer et al. 2021). Fourth, to our knowledge, existing e-SMSPs do not offer real-time educational content tailored to the acoustic environments someone encounters in daily life. Nor are these tailored to an individual’s readiness to take action.

## Hear-aware

The HEAR-aware project aims to address these shortcomings (see Pronk et al. 2020). It covers the development and evaluation of a person-tailored e-SMSP smartphone app aimed at improving HL self-management among 50+ adults who are declining hearing aids or assistive listening devices (ALDs, e.g., a wireless TV listening set), but who are open to self-management support. Improvement of self-management is operationalized as: (1) increased readiness to take action on five target behaviors (TBs) (applying communication strategies, improving emotional coping, seeking social support, taking up hearing aids, taking up ALDs) and (2) improved HL self-management (knowledge, symptoms monitoring management, and emotional management).

This article reports on a nonrandomized intervention study evaluating different aspects of the feasibility of the app prototype intervention in a group of participants with HL. The results will inform a randomized controlled trial in which the app intervention is examined for its effectiveness (Pronk et al. 2020). The present study first focused on the app’s acceptability, linked to the Ecological Momentary Assessment and Ecological Momentary Intervention (EMA; EMI) elements of the app (1). Second, it focused on the stages-of-change (SoC) concept in relation to its measurement (2) and to its potential application as a tailoring method in the app (3).

## Ecological Momentary Assessment and Intervention

EMA is a data capture technique for assessing real-time experiences repeatedly in individuals’ own natural environments (Shiffman et al. 2008). In hearing research, EMA is mostly applied to gain an understanding of the experienced (acoustical aspects) of daily listening of adults with HL, often in the context of hearing aids or other listening devices (for a review, see Holube et al. 2020). The EMA work by Timmer et al. (2017) was the foundation for the HEAR-aware app. In HEAR-aware, users are asked to report on future, present, or recently encountered difficult listening situations via short, automatically prompted, or self-initiated surveys (i.e., EMA, see Methods) with the aim to increase hearing difficulty awareness and thereby motivate a next step in the rehabilitation journey (Timmer et al. 2021). In HEAR-aware, EMA is complemented by micro-level clinical interventions, that is, short pieces of educational material (“snippets”) tailored as much as possible to the user’s reported listening environments, extending it to EMI (McDevitt-Murphy et al. 2018) (see General Methods, Intervention: Four Weeks of App Usage). To our knowledge, this is the first EMA/EMI application in audiological research. An open question addressed in this study is, whether users sufficiently accept frequent participation in EMA surveys followed by educational content (Pronk et al. 2020). See the research questions (RQs) on app acceptability in later sections.

## Stages of Change

Another pillar of the HEAR-aware app is the readiness, or SoC concept derived from the Transtheoretical model of health behavior change (Prochaska & DiClemente 1983; Prochaska & Velicer 1997). This model depicts health behavior change as progress through several SoCs with underlying cognitive or behavioral mechanisms causing attitude shifts. Progress is not necessarily linear; individuals can move in and out of stages and regress. The original model covers five stages, four of which are relevant for HEAR-aware: (1) precontemplation (denial of hearing problems; no plans for action), (2) contemplation (problem awareness and ambivalence regarding the pros and cons of taking action on hearing problems), (3) preparation (change is imminent), and (4) action (taking action on hearing problems). Although the validity of the SoCs is debated (Coulson et al. 2016), studies showed that SoCs have satisfactory construct and concurrent validity, and are predictive of help-seeking, intervention take-up, and outcomes in audiology (e.g., Laplante-Lévesque et al. 2013; Ingo et al. 2016; Saunders et al. 2016; Pronk et al. 2017).

SoC measures traditionally used in audiology are generic, that is, it is not specified what “taking action on hearing” means. In HEAR-aware, readiness is assumed to be TB-specific: an individual can be in different SoCs for different TBs. Hence, generic measures are not considered suitable (Pronk et al. 2020). We therefore adapted two existing, generic SoC scales, that is, the Staging Algorithm (Milstein & Weinstein 2002) and The Line (Rollnick et al. 1999; Tønnesen 2012), into five TB-specific ones. This study examines their test–retest reliability and examines if they indeed measure readiness for different TBs (construct validity). See the RQs on these psychometric properties (numbered from RQ2 onward) in later sections.

Lastly, tailoring intervention content to a recipient’s current SoC is assumed to help move them to higher SoCs (Prochaska et al. 2015). For hearing rehabilitation, Ekberg et al. (2020) piloted clients’ SoC during initial audiology appointments. In contrast, this study explores, whether educational materials tailored to a person’s SoC are better suited to increase readiness. To our knowledge, this study is the first to explore this. See RQ3 later.

In the next sections, General Methods and Results are presented first. Subsequently, Specific Methods and Results per RQ are presented.

## GENERAL METHODS

The design concerned a nonrandomized intervention study to examine different feasibility aspects.

## Sample and Recruitment

The aim was to include n = 50 participants, which is considered the minimum “adequate” sample size for reliability and validity studies (Mokkink et al. 2019). All participants were recruited via a Dutch national hearing aid retailer’s database (Schoonenberg HoorSupport). Participants fulfilling inclusion criteria 1 to 6 below were invited through e-mail or personal letters. An invitation was also posted on the retailer’s Facebook timeline. Researchers checked all criteria during the inclusion phone call:

1. age 50 years or older
2. a minimum pure-tone threshold of 35 dB HL averaged across 1, 2, and 4 kHz in at least one ear
3. visited a Schoonenberg HoorSupport shop for a hearing test appointment or a subsequent intake appointment no longer than 1 year ago, but was not inclined to pursue a hearing aid trajectory
4. owned an e-mail account
5. never tried a hearing aid or ALD
6. still did not want a hearing aid at the time of recruitment
7. owned a smartphone and uses apps
8. was fluent in Dutch
9. was willing to use the app regularly throughout the day for the study period.

All participants provided informed consent. They received €40 for their participation. The Medical Ethics Committee of the Amsterdam University Medical Center (location VUmc), confirmed that ethics approval was not necessary because the Medical Research Involving Human Subjects Act (WMO) did not apply to the study.

## Measurements and Procedure

There were four measurement moments with a 2-week interval between each of them (T0–T3). Table SM1 in Supplemental Digital Content 1 (http://links.lww.com/EANDH/B197) outlines the measurement moments and measures for each of the RQs. Online questionnaires were administered at all four moments and were sent to the participants’ e-mail addresses. They could complete them via their PC, smartphone, or tablet (i.e., not within the app). In the 2-week interval between T0 and T1 no intervention occurred, to facilitate the test–retest reliability analyses (for RQ2.1). App usage data were collected throughout the 4-week usage period (covering T1–T3) and were stored in real time in an online database. Participants received written instructions to download it from Google Play (Android) or App Store (iOS). App start-up and usage were monitored by the researchers in real time via an online content management system. E-mail and telephone assistance were provided in case of technical problems.

## Materials and Methods

### Intervention: Four Weeks of App Usage

The app was hybrid, allowing both Android and iOS smartphone users. It consisted of five main “pages” through which users could navigate via the bottom icons (Figure 1, Supplemental Digital Content 1, panes 1; 3 to 6, http://links.lww.com/EANDH/B197). The aims of the app and instructions for use were explained via the app’s introductory screens, explanation at the top of the main “pages,” and collapsible “?”—icons. The introductory screens appeared automatically when starting up the app for the first time but could be re-accessed at any time.

## EMA Surveys and Listening Situations

Three times a day (10 A.M., 1 P.M., and 7:30 P.M.), a push notification invited participants to fill in short EMA surveys, to report on difficult listening situations. This concerned a clickable pop-up message accompanied by sound and vibration. The times could not be changed to the user’s preference because of technical limitations. There were two kinds of surveys: one for the identification of expected, current, or past difficult listening situations (henceforth: identification surveys), and another for evaluating the previously reported difficult listening situations, which had passed by then (evaluation surveys). Thus, the option to fill out an evaluation survey about a listening situation only appeared after the completion of the identification survey of that particular situation. Note that participants were instructed to continue as usual, and not seek out more difficult listening situations. Participants could also self-initiate surveys instead of responding to a push notification. All survey questions were multiple-choice.

The identification survey comprised three to four questions and took 1 to 2 minutes. Participants selected one of 15 predefined listening situations (e.g., 1-on-1 conversation, small/big group conversations, and watching TV). See Table SM2 in Supplemental Digital Content 1 (http://links.lww.com/EANDH/B197) for all types. Then, users were asked to indicate if background noise was present during this situation. If users reported a listening situation with media, they were also asked to indicate the volume level. Depending on the participant’s answers, the software activated three acoustic labels: (1) type of listening situation, (2) presence of background (yes/no), and (3) presence of loud media volume (yes/no). The app subsequently offered one snippet (see later under “Snippets”) that matched with acoustic labels 1 and 2. When label 3 was activated, a second snippet on potential hearing damage was offered.

The evaluation survey consisted of 8 to 14 questions, depending on the type of listening situation. Completion took 3 to 4 minutes, corresponding to the 3-minute limit suggested by Stone and Shiffman (2002) to reduce response burden and disruption in EMA. Example questions included: “Were face(s) of the speaker(s) visible?” (1: yes, 2: almost always, 3: yes, sometimes, 4: no), “How much effort does/did it take you to hear the sounds you wanted to hear during the listening situation?” (1: I gave up, to 6: no effort), “Did you leave the listening situation earlier than intended because you experienced (some) difficulties hearing?” (1: no, 2: maybe (unconsciously), 3: yes). Another example question is provided in Figure 1 (pane 2), Supplemental Digital Content 1 (http://links.lww.com/EANDH/B197). The survey questions were partly based on Timmer et al. (2017). In addition, users could read short informative “Did you know that”—texts that accompanied certain evaluation survey questions (e.g., “Did you know that seeing the face(s) of your communication partners can help your hearing?” with a short explanation). These were presented via collapsible “i”—icons together with the survey question (see Figure 1, Supplemental Digital Content 1, pane 2, http://links.lww.com/EANDH/B197). Participants were reminded about any unevaluated listening situations via the afternoon and evening notifications, and via red pictograms (see Figure 1, panes 1 and 3, Supplemental Digital Content 1, http://links.lww.com/EANDH/B197). The red pictograms remained visible for as long as situations remained unevaluated. Note that in the same afternoon and evening notification, participants were also asked to add any new situations. Users could also add photos and notes (free text field) to each listening situation. The research team sent reminder e-mails when participants had not added any listening situations in the past 3 (directly after app start-up) or 5 days (later in the usage period).

Both EMA survey forms functioned to elicit awareness of hearing difficulties and the identification survey additionally served to select acoustically tailored snippets. Photographs and notes served to further promote hearing awareness and facilitate evaluation of the listening situations (Saunders et al. 2021). The short informative texts served to educate participants about sound and speech perception.

## Snippets

Snippets (118 in total) were short pieces of educational content and consisted of written text, videos, pictures, sound fragments, or a combination thereof. An example is shown in Figure 1, pane 7, Supplemental Digital Content 1 (http://links.lww.com/EANDH/B197). Snippets varied in length (2 to 10 min. reading/watching time), but most took less than 5 minutes. There were two kinds of snippets: listening situation-induced snippets (81) and standard library snippets (37). The former were offered in response to an entered listening situation (directly after finishing the identification survey) and then became permanently available in the app’s library. Users were invited to directly open the offered snippets, but they could also do that later as they remained retrievable. Each snippet was offered only once. Standard library snippets were available per default in the library.

Where possible, listening situation-induced snippets were acoustically tailored to the entered listening situation. For example, when the situation concerned a “small group (2 to 3 persons) conversation” with “a lot of background noise,” a snippet was offered that fitted the activated listening situation labels (1) “conversation, 2 to 3 persons” and (2) “speech-in-noise” (e.g., snippet “Practicing speechreading”). Around 33% of the listening-induced snippets that were offered had no label and thus, were not offered in a tailored fashion but were offered randomly. The fact that some, and not all, offered snippets were linked to the entered listening situations was explained in the app’s introductory screens. Snippets were supposed to promote TB-specific readiness and an overall sense of self-management.

Each snippet covered one or more of the nine app themes that structured the app’s library (Figure 1, pane 6, Supplemental Digital Content 1, http://links.lww.com/EANDH/B197). Themes and corresponding TBs were: (1) background knowledge of hearing (no TB), (2) communication strategies (TB communication strategies), (3) coping with hearing loss (TB emotional coping), (4) understanding by loved ones (TB social support), (5) hearing at work (no TB), (6) ALDs—to be used without hearing aids (TB ALDs), (7) hearing aids (TB hearing aids), (8) ALDs—to be used with hearing aids (TB hearing aids), and (9) Fun (no TB). The TBs were not visible to the users but the themes were visible.

## Snippet Review Scores

Directly after viewing a snippet, participants were invited to review it on six indicators (pane 8 of Figure 1, Supplemental Digital Content 1, http://links.lww.com/EANDH/B197). This concerned the degree to which they found the snippet: (1) useful, (2) interesting, (3) fun/entertaining, (4) understandable, (5) having the right extent/length, and (6) having a pleasant tone of voice (i.e., appealing). A five-point Likert scale (1 to 5 stars) was used, with higher scores indicating higher satisfaction (Stoyanov et al. 2015). The review request automatically popped up, directly upon closing the snippet. When a participant had reviewed a snippet, it was counted as viewed (for the analyses of RQ1.1).

Initially, reviews were voluntary, but after having noticed that few reviews were returned after 2 weeks of data collection, they were made mandatory via a software update (to allow us to address RQs 1.1 and 3). In addition, a maximum of two short reminder e-mails were sent when participants had reviewed ≤50% of the offered snippets in the past week.

For each library snippet, the participant’s review scores and the average scores of other participants were visible (pane 6 of Figure 1, Supplemental Digital Content 1, http://links.lww.com/EANDH/B197). The latter served to motivate (re-)reading of snippets.

## My Listening Situations and My Statistics

Participants could access an overview of their entered listening situations (My listening situations) with some statistics about them (My statistics), see panes 3; 4, and 5, respectively, of Figure 1, Supplemental Digital Content 1 (http://links.lww.com/EANDH/B197). Participants’ own statistics and those of others were presented for comparison and to promote hearing difficulty awareness (pane 5).

## GENERAL RESULTS

### Recruitment and Sample

Data were collected between July 13, and November 30, 2020. Because of the COVID-19 pandemic in this period, society was partially locked-down, with various government measures being in place. In general, individuals had to keep a ≥1.5 meters distance from nonhousehold members and (large) group gatherings were prohibited or discouraged. In total, 29 participants were included. Reasons for not having opted for a hearing aid earlier are presented in Table SR1, Supplemental Digital Content 2 (http://links.lww.com/EANDH/B198). Twenty-six participated in the full study (all questionnaires, and the app intervention). Three provided data on the T0 and T1 questionnaires (i.e., did not use the app). Characteristics of the 26 app participants are described in Table SR2, Supplemental Digital Content 2 (http://links.lww.com/EANDH/B198). Their mean age was 63.4 years (69% male). Mean HL (better ear, averaged across 1, 2, and 4 kHz) was 34.2 dB HL (SD = 7.6).

## RQ1: APP ACCEPTABILITY

RQs on acceptability, the corresponding analyses, and more details about the app are outlined later. App usage data and the T3 questionnaire were used to answer these RQs (see Table SM1, Supplemental Digital Content 1 (http://links.lww.com/EANDH/B197).

### RQ1.1 Was the Participants’ Compliance With App Usage as Intended?

#### RQ1.1 Measures and Analyses

We focused on the main EMA/EMI elements for compliance: the number of entered and evaluated listening situations and the number of snippets viewed. Compliance criteria were: (1) add at least 1 to 2 (i.e., ≥1.5) listening situations per day, 42 in total; (2) evaluate ≥80% of the added situations; (3a) view ≥80% of offered snippets, (3b) resulting in 33 snippets in total, and (4) view ≥10 standard library snippets. Note that participants were not provided with these or any specific targets, although various app elements (e.g., EMA notifications) did serve as incentives to stimulate daily usage. Descriptive statistics were used. The compliance criteria were based on Timmer et al. (2017) and previous pilot experiences. We held focus groups with potential users in which we presented the app’s main elements and the intended usage, to gauge users’ acceptability.

To explore a possible impact of the COVID lockdown on the number of encountered listening situations, we included a self-designed item in the T3 questionnaire asking if participants had run into difficult listening situations (far) less, equally often, or (far) more frequently due to the COVID lockdown as compared with before.

## RQ1.1 Results

None of the participants met all five compliance criteria, as described in the following paragraphs.

### Listening Situations Added (Compliance Criterion 1)

The 26 participants added 616 listening situations in total (see Table SR3, Supplemental Digital Content 2, http://links.lww.com/EANDH/B198). Most (86%) were self-initiated; 14% were added in response to the morning push notification. Variance in the number of added situations was high and ranged from 0 to 77 (mean, M = 23.7; SD = 16.9). Two participants (8%) met compliance criterion 1 and added 42 listening situations, in line with 1.5 per day. More specifically, three participants (12%) added at least one situation every day, 11 (42%) at least every other day, and 13 (50%) at least every 3 days. The remaining 13 (50%) added 0 to 22 situations (M = 12.8, SD = 5.6) with relatively long passive periods (4 to 20 days no situations added). Twelve participants (46%) reported having come across (far) less difficult listening situations because of the COVID-19 lockdown. This was against three (12%) reporting (far) more, and 11 (42%) reporting no difference.

### Listening Situations Evaluated (Compliance Criterion 2)

The proportion of evaluated situations ranged from 0 to 100% (M = 91%, SD = 0.21). Twenty-two participants (85%) met criterion 2 and reached ≥80% evaluated situations. It was not registered which situations were evaluated in response to a push notification versus which were self-initiated.

### Listening Situation-Induced Snippets Offered and Viewed (Compliance Criteria 3a and 3b)

In total, 648 snippets were offered in response to the 616 added listening situations (32 situations involved media with loud volume, so two and not one snippet was offered). The number of viewed snippets ranged from 0 to 65 per participant (M = 16.3, SD = 14.2). The mean percentage of viewed snippets relative to offered ones was 65% per participant (SD = 31.0; range = 0 to 100). Ten participants (40%) met compliance criterion 3a and viewed ≥80% of the offered snippets. Only 3 participants (12%) met criterion 3b and reached at least 33 viewed snippets.

### Standard Library Snippets Viewed (Compliance Criterion 4)

In total, 211 standard library snippets were viewed, ranging between 0 and 37 across participants (M = 8.1, SD = 10.0). Eight participants (31%) met criterion 4 and reached ≥10 viewed snippets.

## RQ1.2 What is the App’s Overall Usability, Usefulness, and Satisfaction?

### RQ1.2 Measures and Analyses

#### Overall Usability—SUS

The System Usability Scale (SUS) is a validated, 10-item scale providing a global view of users’ perceived usability of a system or device (Brooke 1996). It uses a five-point Likert scale, ranging from strongly disagree (scored 1) to strongly agree (5). An example item is: “I thought the app was easy to use.” The score could range from 0 (lowest) to 100 (highest possible usability) (Brooke 1996). Scores were categorized into 0 to 50 points (awful), 51 to 67 (poor), 68 (OK), 69 to 80.3 (good), and >80.3 points (excellent) (Brooke 1996). The scale showed good internal consistency in our study sample (Cronbach’s α= 0.84).

#### Overall Usefulness—IMI

The value/usefulness subscale of the Intrinsic Motivation Inventory (IMI; Deci & Ryan 1985) was used to determine the app’s usefulness. It covers seven statements rated on a seven-point Likert scale, ranging from not at all true (1) to very true (7). An example item is: “I believe this app could be of some value to me.” The outcome is the mean of all item ratings (possible range 1 to 7, higher scores indicated greater usefulness). The scale showed excellent internal consistency in our sample (Cronbach’s α = 0.94).

#### Overall Satisfaction—IOI-AI and Recommendation Item

We used item 4 of the Dutch-validated International Outcome Inventory-Alternative Intervention (IOI-AI; Kramer et al. 2002): “Considering everything, do you think the app is worth the trouble?” The five response options were: not at all, slightly, moderately, quite a lot, and very much worth it. We also used a recommendation item: “How likely is it that you would recommend the app to other people (family, friends, colleagues)?” Response options could range from 0 (“not at all likely”) to 10 (“extremely likely”). This item was used by Meijerink et al. (2020) and is a measure of client loyalty in marketing research.

### RQ1.2 Results

The mean SUS score was 72.4, indicating “good” overall app usability (Brooke 1996). The variance was substantial (SD = 14.3, range = 32.5 to 100). One participant found the usability awful, nine poor, ten good, and six excellent. The mean IMI score was 4.4 (SD = 1.4, range 1.9 to 7.0), indicating medium overall usefulness, with again a large variance. The mean IOI-AI item 4-score (app satisfaction) showed medium satisfaction (M = 3.1) and large variance: (SD = 1.0, range 1 to 5). One participant found the app “not at all” worth the trouble, six “slightly,” ten “moderately,” seven “quite a lot,” and two “very much.” The mean recommendation item score was 6.0 (SD = 2.6; range 0 to 10) indicating that most were positive (n = 16, score ≥ 6), but a relevant number gave a lower score (n = 10, 38%).

## RQ1.3 What is the Specific Usefulness of the EMA Surveys and the Specific Usefulness and Satisfaction of the Snippets?

### RQ1.3 Measures and Analyses

#### EMA Survey Usefulness

This was measured by a self-designed item administered at T3: “Do you think completing the surveys has provided you with more insight in your hearing?” with four response options: no, not more insight; yes, perhaps a bit more insight; yes, moderately more insight; yes, much more insight.

#### Snippet Usefulness and Satisfaction

Snippet usefulness was measured by review indicator useful (range 1 to 5, see earlier under “Snippet Review scores”) and by one of the EMA evaluation survey questions, that is, “Did the information/tips from the snippet offered earlier today for this particular situation help you in any way?” The four response options were: no, a bit, much, very much. Descriptive statistics were calculated for all snippets lumped together and for different snippet subgroups. Snippet satisfaction was measured by all six review indicators (ranges 1 to 5, see earlier).

### RQ1.3 Results

#### EMA Survey Usefulness

The majority (77%) indicated that filling out the EMA surveys had provided them with “greater insight in their hearing” (12% “much more,” 31% “moderately more,” 34% “a bit more,” 23% “not more insight”).

Table SR4 in Supplemental Digital Content 2 (http://links.lww.com/EANDH/B198) shows the distributions of the EMA evaluation survey question (snippet usefulness) responses and median scores on the six snippet review indicators (snippet usefulness and satisfaction).

#### Snippet Usefulness

With all review snippets lumped together (top row Table SR4, Supplemental Digital Content 2, http://links.lww.com/EANDH/B198), snippets had helped the participants to some extent in 65% of the occasions (“a bit”: 50% of the occasions; ’much’: 13%; “very much”: 2%). In 35% of the occasions, snippets were rated as not helpful (“no”). When stratified for acoustical tailoring, acoustically tailored snippets were found somewhat more useful (“no”: 29% versus ≥“a bit” useful: 71%) than nontailored snippets (“no”: 51% versus ≥“a bit” useful: 49%). When stratified into other snippet subgroups, somewhat differing distributions were found, but the general trend was that most snippets were either rated as not useful (“no”; ranges 19 to 50%), or a bit (ranges 30 to 71%).

#### Snippet Satisfaction

With all review snippets lumped together (top row Table SR4, Supplemental Digital Content 2, http://links.lww.com/EANDH/B198), four review indicators had a median of 4.0. Indicator “fun/ entertaining” only reached 3.0. Acoustically tailored snippets were found somewhat more fun/entertaining (median 3.0) with a more appealing tone (median 4.0) than nontailored snippets (medians 2.0 and 3.0, respectively). When stratified into other snippet subgroups (rather than lumped together), similar medians were found (i.e., 4.0, or 3.0 for “fun/entertaining”). Snippets about ALDs without hearing aids; however, showed a lower median score (3.5) for indicators “useful” and “interesting.”

## RQ2: PSYCHOMETRIC PROPERTIES OF THE 10 NEW SoC MEASURES

### Hypotheses, Methods, and Results

Below, the RQs, our hypotheses, and more details about the SoC measures are provided.

### RQ2.1 What is the Test–Retest Reliability of the New TB-Specific SoC Measures?

We hypothesized good or excellent reliability (intraclass correlation coefficient [ICCs] ≥ 0.6) of the new measures.

### RQ2.2.1 What are the Correlations Between the New TB-Specific SoC Measures, and Between the New TB-Specific SoC Measures and Their Generic SoC Versions (Construct Validity)?

We hypothesized low to moderate correlations (0 to 0.60) between the five TB-specific measures and between each of them and their Generic version, for both the Staging Algorithm and The Line. This would support that they partly measure differing types of readiness.

### RQ2.2.2 How do TB-Specific SoC Constructs Relate to Generic SoC (Construct Validity)?

As an additional way to examine construct validity, we examined which of the five TBs participants thought about when filling out the Generic readiness measures. We hypothesized that the TBs would differ between participants and also the number of TBs. This would confirm that generic measures may measure different types of readiness, depending on the individual.

### RQ2.3 What are the Correlations Between the TB-Specific SoC Measures and Self-Reported Hearing Disability (Construct Validity)?

Consistent with Laplante-Lévesque et al. (2013), we hypothesized that a higher degree of generic readiness would correlate positively with self-reported hearing disability. We also hypothesized that correlations between self-reported hearing disability and each of the TB-specific SoC measures would differ, implying different SoC constructs.

#### RQs 2.1–2.3 Measures

Data for each of the RQs were obtained at T0 and T1 (see Table SM1 in Supplemental Digital Content 1, http://links.lww.com/EANDH/B197). Below, all measures are described. The full SoC questionnaires are provided in Supplemental Digital Content 1, http://links.lww.com/EANDH/B197, pages 6 to 12.

#### Generic SoC—Staging Algorithm Generic

A Dutch version of the 1-item Staging Algorithm adapted for HL (Milstein & Weinstein 2002) was used: “Which of the following statements best describes your view on your current hearing status?” Response options represented the four SoC, and were coded as 0 (precontemplation), 1 (contemplation), 2 (preparation), and 3 (action). Formulation of the response options was adjusted to suit the multiple TBs of the app.

#### Generic SoC—The Line Generic

The original version of The Line (Rollnick et al. 1999; Tønnesen 2012) has been validated and used in various health sciences fields, including hearing research (e.g., Ingo et al. 2017). It asks about perceived importance to “improve hearing now.” We used an adapted version for “readiness to take action” to suit the multiple TBs in the app (“How ready are you to work on your diminished hearing?”). The original discrete 11-point visual analog scale was used. Similar to Ingo et al. (2017), two anchor terms were used, for scores 0 (“not ready at all”) and 10 (“highly ready”).

#### *Tb-Specific SoC—The Line and Staging Algorithm (The 10 New Soc Measures*)

Generic formulations of both The Line and the Staging Algorithm were replaced by the five TBs of the app.

#### Self-Reported Hearing Disability

This was measured using the 28-item Amsterdam Inventory for Auditory Disability and Handicap (Kramer et al. 1995). Summed scores could range from 0 to 74. Higher scores indicated greater hearing disability. Internal consistency in our sample was excellent (Cronbach’s α = 0.90).

#### Self-Reported TBs of Taking Action

This was measured by asking participants what TBs they were thinking about when reading phrases like “taking action on your hearing problems” in the preceding Generic SoC measures. Note that this was asked before any of the TB-specific measures were administered. Participants could tick one or more of the seven predefined response options, including the five TBs, plus “learning to improve my ability to self-manage my hearing problem (e.g., knowing the different treatment options and as such making informed decisions)” and “nothing specific.”

## RQs 2.1–2.3 Analyses

### RQ2.1 Test–Retest Reliability of SoC Measures

T0 and T1 data were used. The 2-week interval between T0 and T1 (with no intervention) was assumed long enough for respondents to forget their answers, and short enough for readiness to remain stable. Reliability of the 10 new TB-specific SoC scales was determined by calculating ICCs and their 95% confidence intervals (95% CIs) (single-measurement, absolute-agreement, two-way mixed-effects model; Koo & Li 2016). ICCs < 0.40 indicated poor reliability, 0.40 to 0.59 fair, 0.60 to 0.74 good, and ≥0.75 excellent reliability (Cicchetti & Sparrow 1981). For comparison, ICCs of the Generic versions of the Staging Algorithm and The Line were assessed.

### RQs 2.2.1, 2.3 Construct Validity: Correlations

Data from T0 were used to determine correlations. Depending on the variables involved, this was either a Pearson correlation coefficient (*r*), Spearman’s rho (*ρ*), or ICC (single measurement, absolute agreement, two-way mixed-effects model).

### RQ2.2.2 Construct Validity: Self-Reported TBs of Taking Action

Data from T0 were used, and descriptive statistics were applied.

## RQs 2.1–2.3 Results

### RQ2.1 Test–Retest Reliability of SoC Measures

The means of The Line Generic were 6.7 (SD = 1.4) and 6.6 (SD = 1.9) at T0 and T1, respectively (see Table 1). According to the Staging Algorithm Generic (Table SR5 in Supplemental Digital Content 2, http://links.lww.com/EANDH/B198), no participants were in precontemplation and few were in the action SoC (n = 4 at both T0 and T1). Most were in contemplation or preparation (n = 14 and n = 11 on T0, respectively). The Line Generic and the Staging Algorithm Generic showed good reliability (ICC = 0.63 and.74, respectively).

**TABLE 1.** Test–retest Reliability (T0 and T1) of SoC-measures, Intraclass Correlation Coefficients (n = 29)

Measure (Possible Range)	M (SD) or Median [25th; 75th Percentile]; Actual Range	M (SD) or Median [25th; 75th Percentile]; Actual Range	ICC	95% CI
	T0	T1		
The Line				
Generic (0–10)	6.7 (1.4); 4–10	6.6 (1.9); 2–10	0.63	0.34–0.81
Communication strategies (0–10)	5.9 [5.0; 7.0]; 0–8	6.2 [4.5; 8.0]; 0–10	0.72	0.49–0.86
Hearing aids (0–10)	6.0 (2.1); 2–10	5.5 (2.7); 0–10	0.53	0.21–0.74
Emotional coping (0–10)	5.1 (2.7); 0–10	6.1 (3.1); 0–10	0.66	0.38–0.83
Social support (0–10)	5.2 (2.2); 0–10	5.6 (3.3); 0–10	0.66	0.39–0.82
ALDs (0–10)	4.7 (2.4); 0–10	4.7 (2.7); 0–8	0.71	0.47–0.86
Staging algorithm				
Generic (0–3)	see Table SR5*; 1–3	see Table SR5*; 1–3	0.74	0.52–0.87
Communication strategies (0–3)	see Table SR6*; 0–3	see Table SR6*; 0–3	0.72	0.49–0.86
Hearing aids (0–3)	see Table SR7*; 0–2	see Table SR7*; 0–2	0.80	0.61–0.90
Emotional coping (0–3)	see Table SR8*; 0–3	see Table SR8*; 0–3	0.51	0.19–0.73
Social support (0–3)	see Table SR9*; 0–3	see Table SR9*; 0–3	0.72	0.48–0.86
ALDs (0–3)	see Table SR10*; 0–3	see Table SR10*; 0–2	0.69	0.44–0.84

published online ahead of print September 6, 2023.

ICCs < 0.40 = poor, 0.40–0.59 = fair, 0.60–0.74 = good, 0.75–1.00 = excellent reliability.

* See Supplemental Digital Content 2, http://links.lww.com/EANDH/B198.

ALDs, Assistive Listening Devices without hearing aids; CI, confidence interval; ICC, intraclass correlation coefficient; M, mean.

For the TB-specific scales of The Line, means ranged between 4.7 (at T0 and T1 for ALDs, SD = 2.4 and 2.7, respectively) and 6.1 (at T1 for Emotional Coping, SD = 3.1). Four of five TB-specific scales showed good reliability (ICCs = 0.66 to 0.74), but for Hearing Aids, it was fair (ICC = 0.53).

In contrast with the Staging Algorithm Generic scale, some participants were in precontemplation and action stages for specific TBs (Tables SR6 to SR10, Supplemental Digital Content 2, http://links.lww.com/EANDH/B198). Exceptions were the Hearing Aids and ALDs scales. These showed a similar picture as of the Generic scale: no participants in the action SoC for ALDs and Hearing Aids and few in precontemplation for Hearing Aids. Three TB-specific Staging Algorithm scales showed good reliability: ALDs, Social Support, and Communication Strategies (ICCs = 0.69 to 0.72). Reliability was excellent on the Hearing Aids scale (ICC = 0.80) and fair on the Emotional Coping scale (ICC = 0.51).

### RQ2.2.1 Construct Validity: Correlations Between Each of the SoC Measures

Rows 1 and 7 of Table SR11 (Supplemental Digital Content 2, http://links.lww.com/EANDH/B198) present correlations between each of the TB-specific scales of The Line (upper part) or the Staging Algorithm (lower part), as well as between them and their Generic scales, respectively. The Generic scales were significantly correlated with Communication Strategies (The Line: *ρ* = 0.47) and Hearing Aids (The Line: *r* = 0.44, Staging Algorithm: ICC = 0.25). Most other TB-specific scales were either not significantly correlated with each other (The Line), or moderately (ICC < 0.60; Staging Algorithm Emotional Coping with Communication Strategies and ALDs). An exception was the correlation between the Social Support and Communication Strategies scales, which was relatively strong (≥0.60), both for The Line (*ρ* = 0.74) and the Staging Algorithm (ICC = 0.70).

### RQ2.2.2 Construct Validity: Self-Reported TBs of Taking Action

The participants (n = 29) had a mean of 1.5 TBs in mind when they reported their readiness to take action on their hearing problems, as measured via the Generic SoC measures (SD = 0.9, range 0 to 3). The TBs that most participants thought about were getting hearing aids (n = 20), learning communication strategies (n = 13), and getting ALDs (n = 7). Improving emotional coping (n = 3) and seeking social support (n = 1) were reported less often. Two respondents reported not having any specific TB in mind when filling out the Generic readiness measure. Of that reporting to have had to get hearing aids in mind (n = 20), eight only reported this TB, six, in addition, reported learning communication strategies, three additionally reported both learning communication strategies and getting ALDs, two additionally reported getting ALDs, and one additionally reported emotional coping. Lastly, 14 reported to have “improving self-management” in mind, of which 12 did so in addition to thinking about at least one of the five TBs.

## RQ2.3 Construct Validity: Correlations Between SoC Measures and Self-Reported Hearing Disability

In the last column of Table SR11 (Supplemental Digital Content 2, http://links.lww.com/EANDH/B198), the correlations with self-reported hearing disability are shown. Only the Hearing Aids scale was significantly correlated with self-reported hearing disability, both for The Line (*r* = 0.39) and the Staging Algorithm (*ρ* = 0.45).

### RQ3 Stages-of-Change Tailoring of Snippets

#### RQ3 Hypothesis, Methods, and Results

With RQ3, we explored whether snippets tailored to the participant’s SoC would suit the individual’s needs better than nontailored materials, which would imply that SoC-tailored materials would be better suited to increase readiness to take action (Prochaska et al. 2015).

As mentioned, each snippet had content addressing one or more app themes, each of which in turn corresponded to a TB. For RQ3, each snippet was a priori assigned a main TB (in case the snippet addressed only one TB, this automatically became the main TB) and a main SoC that each snippet was deemed most suitable for. Both the TB and SoC assignments were done by the same researcher, who used literature study and a self-designed summary document to assign SoC. SoC assignments of the first 15 snippets, and of any unclear snippets were discussed with a second researcher. Table SM3 of Supplemental Digital Content 1, http://links.lww.com/EANDH/B197 shows the distribution of the main SoCs and TBs across snippets. Note that the snippet’s main TB and SoCs were not visible to the participants. Examples of two snippets that both addressed TB communication strategies, but were assigned two different main SoCs are provided, in Supplemental Digital Content 1 (pages 5 to 6) (http://links.lww.com/EANDH/B197).

In addition to having assigned the TB-specific SoC for each snippet, we assumed that each participant would be in a particular SoC for a particular TB (SoC_person_, see later under “RQ3 Measures”). These two elements allowed us to determine whether there was agreement between participant’s SoC and the snippet’s SoC, or disagreement, and how far they were apart then (i.e., SoC_difference_, see later under “RQ3 Measures”). Lastly, as mentioned, users could indicate their snippet satisfaction on six indicators. We used two of them to operationalize “satisfaction,” to address the last RQ:

### RQ3 What is the Relationship Between Soc_difference_ and the Participant’s Satisfaction (Review Indicators “Interesting” and “Useful”) with the Snippet?

We hypothesized that lower SoC_difference_ (so less disagreement, or in other words, more agreement between the snippet’s and participant’s SoC) would be linked to higher snippet satisfaction scores.

## RQ3 Measures

### Snippet Satisfaction

The two scores of review indicators “useful” and “interesting” (scores 1 to 5) were used.

### SoC_difference_ and Associated Measures

To calculate SoC_difference_, SoC_snippet_, and SoC_person_ were used. These are explained first. SoC_snippet_. SoC_snippet_ denoted the main SoC the reviewed snippet tapped into (see earlier). This variable was coded 0 (precontemplation), 1 (contemplation), 2 (preparation), and 3 (action).

SoC_person_. SoC_person_ included the participant’s TB-specific SoC as measured by the TB-specific Staging Algorithm. We chose the Staging Algorithm because The Line has no validated cutoff points. SoC_person_ was coded similarly to SoC_snippet_ (0 to 3).

Each snippet satisfaction rating was linked either to the participant’s SoC_person_ as measured at T1 or T2. Thus, for snippets reviewed between T1 and T2, participants’ T1 SoC_person_ score was used in the analyses, whereas for snippets reviewed between T2 and T3, their T2 SoC_person_ score was used.

SoC_difference_. SoC_difference_ was calculated as SoC_snippet_− SoC_person_, reflecting the difference between the participant’s SoC (SoC_person_), and the SoC the snippet tapped into (SoC_snippet_) for the same TB. For snippets without a specific TB, the participant’s Generic SoC Staging Algorithm score was used for SoC_person_. Scores for SoC_difference_ could range from -3 (SoC_snippet_ was much lower than SoC_person_) to 3 (SoC_snippet_ was much higher than SoC_person_). Zero indicated full SoC correspondence and was set as the reference value. SoC_difference_ was treated as a categorical variable as it showed no linear relationships with “useful” and “interesting.”

## RQ3 Analyses

The relationships between the participants’ snippet satisfaction and SoC_difference_ were assessed using two separate linear mixed models which are robust for non-normally distributed outcomes and for missing values at random. The review indicators “interesting” and “®useful” were entered as continuous outcome variables and the independent variable of interest was SoC_difference_. SoC_snippet_ was included as a corrective factor. To illustrate, the conceptual equation for the outcome “interesting” was: snippet satisfaction_interesting_ = SoC_difference_ + SoC_snippet_. The overall relationship was considered statistically significant if the coefficient of SoC_difference_ was significantly associated with satisfaction (*p* < 0.025). A Bonferroni correction was applied because of the two satisfaction outcomes (0.05/2). Post hoc analyses were used to determine the statistical significance of the estimates for the five individual nonzero SoC_difference_ values compared with an SoC_difference_ of 0 (*p* < 0.01, that is, also Bonferroni-corrected: 0.05/5).

## RQ3 Results

Table SR12 in Supplemental Digital Content 2 (http://links.lww.com/EANDH/B198) shows the descriptives of SoC_person_ and SoC_snippet_. Table 2 shows the results of the two linear mixed models.

**TABLE 2.** Relationships between SoC_difference_ and Snippet Satisfaction Ratings “Interesting” and “Useful” (n = 25)

	Interesting			Useful		
		Estimate (95% CI)	*p*		Estimate (95% CI)	*p*
Number of observations^†^	SoC_difference_*	—	**0.006**	SoC_difference_*		0.033
19 (6; 16)	−3	0.85 (0.37 to 1.32)	**<0.001**	−3	0.71 (0.21 to 1.21)	**0.005**
69 (15; 34)	−2	0.03 (−0.21 to 0.28)	0.783	−2	0.15 (−0.10 to 0.41)	0.239
176 (22; 74)	−1	0.13 (−0.06 to 0.33)	0.185	−1	0.07 (−0.13 to 0.28)	0.489
260 (24; 73)	0 (reference)	—	—	(reference)	—	—
85 (18; 29)	1	−0.10 (−0.34 to 0.14)	0.426	1	−0.20 (−0.45 to 0.05)	0.125
26 (9; 14)	2	−0.33 (−0.71 to 0.06)	0.095	2	−0.33 (−0.73 to 0.07)	0.101

Both models were corrected for SoC_snippet_. Statistically significant associations are indicated in **bold**. *p* < 0.025 was statistically significant for the overall association and *p* < 0.01 for the post hoc tests (Bonferroni-corrected).

* Potential range: −3 (SoC_snippet_ was much lower than SoC_person_) to 3 (SoC_snippet_ was much higher than SoC_person_).

† Number of unique participants; unique snippets are presented between brackets.

—, not applicable; SoC, Stage of Change.

For indicator “interesting,” a significant overall association with SoC_difference_ was found (*p* = 0.006). Post hoc testing showed that only for snippets with a much lower SoC than the participant (i.e., SoC_difference_ = −3) there was a significant difference with the reference (*p* < 0.001). Thus, participants rated snippets with a much lower SoC than their personal SoC (i.e., a three-stage difference) 0.85 points more “interesting” as compared with snippets with a SoC similar to their personal SoC (i.e., full-stage agreement). Although not statistically significant post hoc, the direction of the effect also applied to SoC_difference_ values −2 and −1. Thus, the further away the snippet’s SoC was from the participant’s SoC (i.e., the relatively lower the snippet’s SoC was) the more interesting the participant rated the snippet. This direction was contrary to our hypothesis.

For snippets with a higher SoC than the participant (i.e., positive values of SoC_difference_), the direction was reversed and in the expected direction. Now, snippets with a higher SoC than the participant was associated with lower “interesting” scores. However, these associations were not significant post hoc (*p* ≥ 0.095).

For “useful” there was a more or less similar trend in the estimates visible as for “interesting.” However, the overall association for “useful” was borderline significant (*p* = 0.033).

## DISCUSSION

This study reports on the findings of an intervention study among adults who had been recently diagnosed with HL and declined a hearing aid trajectory but were open to an e-SMSP in the shape of the 4-week HEAR-aware app intervention. Key uncertainties were examined and related to: the app’s acceptability (RQ1), the psychometric properties of ten newly developed TB-specific SoC scales (RQ2), and the potential of using the SoC concept for person-tailoring of educational materials (RQ3). Later, the principal results and study limitations are discussed.

### (1) Acceptability of the HEAR-aware App

Only 8% of participants met the intended usage of adding ≥1.5 listening situations a day, resulting in 42 snippets in total across the 4-week intervention period. The average participant added about half of that volume (i.e., M = 23.7). Although this number might be an underestimation as half of the sample underreported difficult listening situations because of the COVID-19 lockdown circumstances, the observed compliance seems generally in line with other EMA research (Holube et al., 2020). In contrast, Timmer et al. (2017) found a much higher average number of 2.85 added situations a day, with similar EMA questions in a sample of adults with mild HL. Comparison with that study is however difficult, because it concerned a highly motivated sample, had a different goal (scientific data collection and not self-management support), and the participant burden was relatively low (collection of singular and not two-stage EMA surveys, for two and not four weeks). We found some supporting evidence for the latter, as HEAR-aware participants added somewhat more listening situations in the first two weeks (M = 13.8) than in the second (M = 10.8, *p* = 0.007).

Low satisfaction with the snippets’ usefulness may have limited the compliance somewhat (see further later). In addition, we found anecdotal evidence for other reasons directly or indirectly leading to underreporting of listening situations, in the free text field at the end of the T3 questionnaire (e.g., “I became sloppy with filling out surveys because they became repetitive,” and “The app gave no direct feedback on my responses in the surveys”). In contrast, the relative usage compliance criteria 2 and 3a (i.e., ≥80% of listening situations evaluated and listening situation-induced snippets viewed, respectively) showed higher satisfactory acceptability. Compliance rates for these criteria were 85% and 40% of the participants, respectively, suggesting that once participants had added listening situations and received snippets, they were generally inclined to take the next steps (i.e., evaluation of listening situations, and viewing the offered snippets). It should be noted that there was some uncertainty about whether snippets were actually viewed as we used a proxy measure of viewing (i.e., it was only certain that the snippet was reviewed, and thus, opened).

#### Overall Usability, Usefulness, and Satisfaction (RQ1.2)

Overall usability (SUS) of the HEAR-aware app was good, and overall usefulness (IMI) was satisfactory, albeit with large variances. Philips et al. (2018) found smaller variances for their CI tablet app, despite a limited sample size. A similar pattern (reasonable median score, large variance) was found for the recommendation item. The IOI-AI-satisfaction item showed a more positive outcome: 73% found the app “moderately” to “very much worth the trouble.” We conclude that the app’s overall usability, usefulness, and satisfaction were reasonably positive. Nonetheless, the large variances indicated room for improvement.

#### Specific Usefulness—EMA Surveys (RQ1.3)

The majority (77%) of the participants indicated that filling out the EMA surveys had provided them with greater insight into their hearing. This is encouraging, especially because hearing difficulty awareness is considered an important outcome in HEAR-aware. As pointed out earlier (Pronk et al. 2020), the question is, which intervention dosage (number of EMA surveys with listening situation-induced snippets, see later) gives an effect on the targeted outcomes and is acceptable? Future research is needed to shed more light on this.

#### Specific Satisfaction and Usefulness—Snippets (RQ1.3)

Satisfaction with the snippets as measured by the six review indicators was considered sufficient (median scores of 3.5 or 4 of 5). The median snippet usefulness review indicator score was 4. In contrast, usefulness was rated less positive on the EMA evaluation survey question: about one-third of the snippets were not found useful at all (35%). However, the EMA survey asked about the direct helpfulness of the offered information/tips to the particular listening situation, whereas the snippet review score indicated a more general usefulness of the snippet. In line with this, the snippets that were acoustically tailored to the listening situation were rated somewhat more useful on the EMA survey question (at least a bit useful: 71%) than the nonacoustically tailored snippets (at least a bit useful: 49%). Although it was explained in the app’s introductory screens that not all offered snippets were linked to the entered listening situation, the mere fact that the snippets were offered directly after an entered listening situation may have given this impression nonetheless. Anecdotal evidence from the T3 questionnaire supported this: “The predefined connection between the snippets and listening situations was not always useful.” In turn, this may explain some of the suboptimal results found for both usefulness parameters. Lastly, it should be noted that both parameters were administered directly after viewing the snippets, whereas some information may have needed more time to sink in, or may have concerned an unconscious process.

#### Target Group of HEAR-aware

A last acceptability-related finding worth mentioning is that the adults reached with HEAR-aware were 5 to 9 years younger and had 7 to 10 dB HL better hearing thresholds than participants in two previous intervention studies (Meijerink et al. 2020 and Pronk et al. 2017, respectively). These studies applied similar inclusion criteria, recruited from the same hearing aid retailer, but offered hearing aid fitting plus a PC-based e-SMSP, and hearing aid fitting only as intervention, respectively. These differences suggest that adults with HL who do not accept a hearing aid (and would otherwise remain unserved), can be reached at an earlier moment in their hearing help-seeking journey through e-SMSPs, especially by means of a smartphone app.

### (2) Psychometric Properties of the 10 New TB-Specific SoC Measures

#### Test–Retest Reliability (RQ2.1)

In general, the reliability of the new TB-specific SoC scales was satisfactory (≥0.60) and comparable to the Generic scales. Two TB-specific scales however scored somewhat lower and strictly may be considered insufficient (The Line Hearing Aids, ICC = 0.53; Staging Algorithm Emotional Coping, ICC = 0.51). Although the reliability findings are encouraging, some caution is needed when interpreting the findings. The range for some of the scales was limited. Similar to the Staging Algorithm Generic, no to few participants were in the precontemplation and action SoCs for two TB-specific Staging Algorithm scales (precontemplation for TB Hearing Aids; action for TBs Hearing Aids and ALDs). Also, the sample size was 29, which is on the border of being inadequate (n < 30) and doubtful (n = 30 to 49) for reliability and validity testing according to Mokkink et al. (2019).

#### Construct Validity (RQs 2.2.1, 2.2.2, 2.3)

The present study’s results on self-reported TBs of taking action (RQ2.2.2) showed that the Generic SoC measures assess readiness on different, and often multiple TBs per individual. This is consistent with earlier work (Pronk et al. 2020) and with our hypothesis. It supports that readiness should be measured in a TB-specific fashion. Although a generic measure may give an overall impression of a person’s readiness to do “something” about their HL, it is troublesome that the underlying construct is likely to differ across individuals, and possibly also within an individual over time (longitudinal measurements). Communication strategies and hearing aids were the TBs thought of most frequently when participants read about “taking action on hearing.” This was in line with our finding that these were the only two TB-specific scales significantly correlated with The Line Generic (both TBs) and the Staging Algorithm (Hearing Aids only).

In line with our hypothesis, most TB-specific scales were not or moderately correlated with each other (*r* < 0.60), thus measuring largely distinct types of readiness. In contrast, the TB-specific Social Support and Communication Strategies scales were strongly correlated (*ρ* = 0.74 and ICC = 0.70). A strong social circle may make the person with HL feel more confident or comfortable practicing communication strategies. This seems consistent with studies showing that persons with HL with strong social support generally have better outcomes (e.g., Kramer et al., 2005; Hickson et al., 2007; Preminger & Rothpletz 2016). It should be noted that we measured *readiness* for and not the actual experience of social support. Nonetheless, it seems plausible that persons who already experienced some social support also report higher readiness for seeking such support for their hearing problems.

Contrary to Laplante-Lévesque et al. (2013) and to our hypothesis, we did not find a significant correlation between self-reported hearing disability and any of the Generic SoC scales. It is interesting that we observed a significant correlation with the Hearing Aids scales, but -unexpectedly - not with the other TB-specific scales. We speculate that the absence of a correlation between self-reported hearing disability and generic readiness was because about half of the sample thought about hearing aids while filling out the Generic scales, in addition, thought about other TBs, and the latter thus may have been more top-of-mind than in Laplante-Lévesque et al. (2013). As readiness for these other TBs was not significantly correlated with self-reported hearing disability in our sample, this diluted the correlations for the Generic scales. It remains unclear why the other TB-specific scales were not correlated with self-reported hearing disability, as we expected partly similar underlying processes occurring for hearing aids (higher levels of self-reported disability tend to be correlated with more severe levels and longer duration of HL, and go hand in hand with more acceptance and readiness to take action). In any case, the correlation between self-reported hearing disability and readiness to take up hearing aids is in line with evidence showing a strong relationship with hearing help-seeking steps including hearing aid uptake (Knudsen et al., 2010).

### (3) Stages-of-Change Tailoring of Snippets

Unexpectedly, snippets with a lower SoC than that of the participant (i.e., negative SoC_difference_ scores), were rated as more interesting than matching snippets. Although borderline significant, a similar result was found for “useful.” In contrast, we expected lower satisfaction scores with larger SoC-differences. We speculate that educational information with a “too low SoC” for a person may have been considered confirmative and reassuring (possibly boosting self-efficacy). Alternatively, the information may have simply been easier to understand and relate to. Both mechanisms could have subsequently translated into positive snippet satisfaction scores.

There were few participants with an SoC_difference_ score of −3, and the post hoc analyses showed no significant differences for the −2 and −1 scores. Despite the uncertainty this introduces, the overall direction of the effect for the negative SoC_difference_ scores was significant and therefore seems robust. It should be mentioned that SoC_difference_ score −3 only included participants with high readiness, that is, in the action SoC (see Table SR12, Supplemental Digital Content 2, http://links.lww.com/EANDH/B198). Furthermore, neither persons in the action SoC nor the preparation SoC were represented in the positive SoC_difference_ scores, as no snippets with an action SoC or higher readiness were available in the app. Therefore, it remains unclear if the confirmative and reassuring effect hypothesized earlier plays a role in individuals with a high readiness (preparation, action) only or also in those with lower readiness (contemplation).

The overall association between SoC_difference_ and both satisfaction indicators showed a more or less similar trend in the estimates, including the association for positive SoC_difference_ scores (i.e., snippets with a higher SoC than the participant). So, both for “interesting” and “useful,” the direction of this relationship was in the expected direction, that is, participants reported lower satisfaction when the SoC of the snippet was “too high” for them. However, as the overall association was not statistically significant for “useful,” this result remains unsure.

In summary, part of the SoC-tailoring results were unexpected and called for further study. Moreover, there are many factors possibly influencing why someone finds educational materials interesting or useful and these may have somehow confounded with SoC_difference_ (via SoC_person_ or SoC_snippet_). Lastly, we assumed that persons would find snippets more interesting and useful when corresponding to their SoC, but this assumption may not be true. Readiness-tailored educational messages may still impact a person’s attitudes and beliefs (and thus bring about movement in SoCs), despite the message not being rated interesting or useful.

### Conclusions

Overall satisfaction, usability, and usefulness of the HEAR-aware app were reasonable to good, indicating sufficient acceptability. The large variance between participants in these outcomes, and the suboptimal compliance to the app’s EMA/EMI elements indicated room for improvement for part of the participants. The 10 new TB-specific SoC scales generally showed fair-to-excellent test–retest reliabilities, and sufficient construct validity, indicating their promise for use in future studies. The results of this study may inform future research on how to tailor educational app materials to a person’s SoC.

## ACKNOWLEDGMENTS

We acknowledge everywhereIM for their role in the development and implementation of the HEAR-aware app in the study. We thank Schoonenberg HoorSupport for allowing the use of “HoorSupport,” and VeiligheidNL (formerly Nationale Hoorstichting) for the use of the book “101 vragen over horen,” for snippet content.

HEAR-aware concerns a collaboration project co-funded by Sonova AG (Switzerland), Schoonenberg HoorSupport (the Netherlands), and the PPP Allowance made available by Health~Holland, Top Sector Life Sciences and Health, to stimulate public-private partnerships.

## Supplementary Material




