# Supplementary material for: Feasibility of the HEAR-aware App for Hearing Loss Self-Management: A Nonrandomized Intervention Study to Examine Intervention Acceptability and the Stages-of-Change Concept
Source: Ear Hear. 2023 Sep 6;45(1):186–97. doi: 10.1097/AUD.0000000000001414 (PMC10718215; doi:10.1097/AUD.0000000000001414)

Supplemental Digital Content 1: Methods

Content:

- **TABLE SM1.** *Overview of the Study's Measurements and Research Questions*
- **TABLE SM2.** *The Types of Listening Situations that Users Could Add via the EMA Identification Survey of the HEAR-aware App*
- **TABLE SM3.** *Number of Snippets with the Main Stages of Change and Main Target behaviors they Tapped into*
    - **Two Example Snippets with Different Main Stages of Change**
- **Stages of Change Measures**
- **Fig. 1.** *Screenshots of the App's Main Pages.*

TABLE SM1. *Overview of the Study's Measurements and Research Questions*◊

Constructs and measures	T0	T1	T2	T3	Data collection method	RQ◊
**Descriptive sample characteristics**	X				Online questionnaire	-
**App acceptability**<br>App usage and compliance<br>- Added and evaluated listening situations<br>- Influence of COVID lockdown on number of listening situations<br>- Viewed snippets	 	 <br>X<br><br>X	 <br>X<br><br>X	 <br>X<br>X<br>X	 <br>The app*<br>Online questionnaire<br>The app*	1.1
**App acceptability**<br>Overall app usability:<br>- SUS<br>Overall app usefulness:<br>- IMI<br>Overall app satisfaction:<br>- IOI-AI item 4<br>- Recommendation item				 <br>X<br><br>X<br><br>X<br>X	 <br>Online questionnaire<br><br>Online questionnaire<br><br>Online questionnaire<br>Online questionnaire	1.2
**App acceptability**<br>Specific app usability:<br>EMA survey usefulness:<br>- Item in the T3 questionnaire				 <br><br>X	 <br><br>Online questionnaire	1.3
Snippet usefulness:<br>- Snippet review indicator 'useful'<br>- EMA evaluation survey item on snippet usefulness		 X<br>X	 X<br>X	 X<br>X	 The app*<br>The app*	1.3
Snippet satisfaction:<br>- All 6 review indicators		 X	 X	 X	 The app*	1.3
**Psychometric properties of SoC measures**<br>- Staging Algorithm Generic<br>- Staging Algorithm TB-specific<br>- The Line Generic<br>- The Line TB-specific	 X<br>X<br>X<br>X	 X<br>X<br>X<br>X			 Online questionnaire<br>Online questionnaire<br>Online questionnaire<br>Online questionnaire	2.1 - 2.3

- Self-reported hearing disability<br>- Item asking about the TBs that the participant thought about when reading generic SoC measures	X<br>X				Online questionnaire<br>Online questionnaire	
**<u>SoC tailoring of snippets</u>**						3
- Staging Algorithm TB-specific (for SoC_{person})		X	X		Online questionnaire	
- Staging Algorithm Generic (for SoC_{person})		X	X		Online questionnaire	
- Main SoC and main TB per snippet (for $SoC_{snippet}$)		X	X	X	The app*	
- Snippet review indicators 'useful' and 'interesting'		X	X	X	The app*	

EMA, Ecological Momentary Assessment; IOI-AI, International Outcome Inventory- Alternative Intervention; IMI, Intrinsic Motivation Inventory; RQ, research question; TB, target behavior; SoC, Stage of Change; SUS, System Usability Scale; -, not applicable.
*Data of 'The app' were collected throughout the 4-week intervention period, which started at T1 and ended at T3 (indicated in the table with an 'X' at T1, T2, and T3).
◊ The RQs were:

1. App acceptability
RQ1.1 Was the participants' compliance to app usage as intended?
RQ1.2 What is the app's overall usability, usefulness, and satisfaction?
RQ1.3 What is the specific usefulness of the EMA surveys and the specific usefulness and satisfaction of the snippets?

2. Psychometric properties of SoC measures
RQ2.1 What is the test-retest reliability of the 10 new TB-specific SoC measures?
RQ2.2.1 What are the correlations between the new TB-specific SoC measures, and between the new TB-specific SoC measures and their generic SoC versions (construct validity)?
RQ2.2.2 How do TB-specific SoC constructs relate to generic SoC (construct validity)?
RQ2.3 What are the correlations between the TB-specific SoC measures and self-reported hearing disability (construct validity)?

<u>3. SoC tailoring of snippets</u>
RQ3: What is the relationship between SoC-difference and the participant's satisfaction with the snippet?

TABLE SM2. *The Types of Listening Situations that Users Could Add via the EMA Identification Survey of the HEAR-aware App*

1. (Video)calling
2. 1-on-1 conversation
3. Group conversation, small (with 2-3 other persons)
4. Group conversation, big (with 4 or more other persons)
5. Lecture/ presentation
6. Sports instruction
7. Watching TV – On TV/PC/laptop
8. Watching clips – On smartphone
9. Radio interview / podcast / audiobook
10. Music
11. Cycling *(e.g., hearing other traffic approaching, hearing bicycle bells ringing/ cars honking)*
12. Driving the car *(e.g., hearing other cars honking, emergency vehicles sirens)*
13. Using public transport *(e.g., hearing check-in/check-out signals, public address systems)*
14. Walking *(e.g., hearing other traffic approaching, hearing bicycle bells ringing/ cars honking)*
15. Nature sounds *(e.g., hearing birds singing, leafs rustling, sounds of the sea)*
16. Other, namely…

EMA: Ecological Momentary Assessment

TABLE SM3. *Number of Snippets with the Main Stages of Change and Main Target Behaviors they Tapped into*

	Main Stage of Change			
	Precontemplation	Contemplation	Preparation	Total
**Main target behavior**				
Communication Strategies	0	1	8	9
Emotional Coping	1	4	0	5
Social Support	0	2	1	5
ALDs without hearing aids	0	0	3	3
ALDs with hearing aids	0	3	1	4
Hearing Aids	3	20	6	29
No target behavior	28	36	1	65
Total	32	66	30	118

ALDs, Assistive listening devices.

Explanation: Two Snippets with Two Different Main Stages of Change

The snippet shown in pane 7 of Figure 1 (see end of document) concerned main TB communication strategies and main SoC preparation. It contained two videos. The first one showed a female trainer who demonstrated the added value of using hand gestures. The second one showed a role model (person with hearing problems) demonstrating subsequently ineffective and effective communication strategies in a daily life situation. Hands-on tips and instructions were provided via the female trainer and accompanying text. Thus, these snippet elements aimed to underline important pros of applying effective communication strategies and promote self-efficacy, respectively. Thereby, it was deemed to especially serve persons in preparation, already planning to apply strategies in the near future. An example of a communication strategy snippet assigned to the adjacent, lower, contemplation SoC was the text-based snippet ‘The effects of hand gestures’ (not shown). It concerned an article written in a popular-scientific style, discussing the pros of communication partners using hand gestures when speaking to persons in general, but also when speaking to a person with hearing problems. The article suggested the reader to consider sharing the article and/or discussing the topic with a loved one. These snippet elements were assumed to mainly suit persons who had contemplating thoughts about asking significant others to adjust their communication style somewhat. The snippet meant

move the person towards taking a first step, which would move them to the adjacent SoC, i.e., preparation.

Stages of Change Measures

Below, the generic and target-behavior specific Stage-of-Change measures (adapted versions of the Staging Algorithm and The Line) that were used in the HEAR-aware study are provided. They were freely translated into English, from the Dutch version used in HEAR-aware. The original, Dutch versions can be obtained via the authors.

See Table SM1 above. All SoC measures below were administered via online questionnaires, on both T0 and T1. These data were used in RQs 2.1-2.3 (to examine the measures' psychometric properties). In addition, all Staging Algorithm scales were also administered on T2. The Staging Algorithm data on T1 and T2 were used for the analyses of RQ3 (to determine SoC_{person}, to be able to examine the potential of SoC-tailoring of the snippets).

Staging Algorithm- Generic

Which of the following statements best describes your view of your current hearing status? Tick this statement.

- ❑ I do not think I have any problems involving my hearing, and therefore I do not have to do anything about it.
- ❑ I think I have problems involving my hearing. However, I am not yet ready to take any action to address them now, but I might do so in the future.
- ❑ I know I have problems involving my hearing, and I intend to take action to address them soon.
- ❑ I know I have problems involving my hearing, and I am taking action to address them now.

The Line - Generic

How ready are you to work on your diminished hearing?

Please choose a number between 0 and 10 by moving the slider.

0	1	2	3	4	5	6	7	8	9	10
Not ready at all										Highly ready

Staging Algorithm- Target-behavior specific measures (i.e., consecutively: Communication Strategies, Emotional Coping, Social Support, Hearing Aids, Assistive Listening Devices)

For the following 5 questions, we would like to know how you currently feel about taking **specific actions** for your diminished hearing. Please tick the statement that fits best.

1. Which of the following statements best describes your current view on **communication strategies***?
   Think of: consciously applying speech reading (lip reading, reading facial expressions), purposefully choosing a location in the room to sit or stand so that you can understand your communication partners well, asking others to articulate well or speak more slowly.
   - ❑ I don’t think my hearing problem is such that communication strategies would be of any help
   - ❑ I think I have a hearing problem where communication strategies may be helpful. I am just not ready yet to put them into practice now, but I might be in the future.
   - ❑ I know I have a hearing problem where communication strategies could be helpful and I intend to put them into practice soon.

- ❑ I know I have a hearing problem where communication strategies can be helpful, and therefore I am already putting them into practice.

2. Which of the following statements best describes your current view on **working on your feelings about your diminished hearing***?
   *Think of learning to cope with the challenges or problems around your diminished hearing (learning to accept, to decrease any negative feelings)
   - ❑ I don't think my hearing problem is such that working on my feelings would be of any help.
   - ❑ I think I have a hearing problem where working on my feelings may be helpful. I am just not ready yet to put them into practice now, but I might be in the future.
   - ❑ I know I have a hearing problem where working on my feelings could be helpful, and I intend to do this soon.
   - ❑ I know I have a hearing problem where working on my feelings is helpful, and therefore I am already working on this.

3. Which of the following statements best describes your current view on **involving others in your diminished hearing***?
   * Think of sharing any challenges and problems around your hearing with others. This can, but doesn't necessarily need, to be about sharing feelings.
   - ❑ I don't think my hearing problem is such that involving others would be of any help.
   - ❑ I think I have a hearing problem where involving others may be helpful. I am just not ready yet to put them into practice now, but I might be in the future.
   - ❑ I know I have a hearing problem where involving others could be helpful, and I intend to do this soon.
   - ❑ I know I have a hearing problem where involving others can be helpful, and therefore I am already working on this.

4. Which of the following statements best describes your current view on **hearing aids**?
   - ❑ I don't think my hearing problem is such that hearing aids would be of any help.
   - ❑ I think I have a hearing problem where hearing aids may be helpful. I am just not ready yet to try them now, but I might be in the future.
   - ❑ I know I have a hearing problem where hearing aids could be helpful, and I intend to do try them soon.
   - ❑ I know I have a hearing problem where hearing aids can be helpful, and I have already scheduled or had a hearing aid trial / am already using hearing aids.

5. Which of the following statements best describes your current view on **assistive listening devices***?

   * For example, think of amplified headphones for the TV, a doorbell amplifier, or a vibrating alarm clock.
   - ❑ I don't think my hearing problem is such that assistive listening devices would be of any help.
   - ❑ I think I have a hearing problem where assistive listening devices may be helpful. I am just not ready yet to try them now, but I might be in the future.
   - ❑ I know I have a hearing problem where assistive listening devices could be helpful, and I intend to do try them soon.
   - ❑ I know I have a hearing problem where assistive listening devices can be helpful, and I have already scheduled or had a trial period / am already using an assistive listening device.

The Line - Target-behavior specific measures (i.e., consecutively: Communication Strategies, Emotional Coping, Social Support, Hearing Aids, Assistive Listening Devices)

For the following 5 questions, we would like to know *how ready you are* to take specific actions for your diminished hearing. Please note that it again concerns how you feel *at the moment*.

For each question, choose a number between 0 and 10 by moving the slider. 0 indicates: ‘Not ready at all’ and 10 indicates: ‘Highly ready’.

1. How ready are you to apply **communication strategies*** in your daily life?

   * For example, think of: consciously applying speech reading (lip reading, reading facial expressions), purposefully choosing a location in the room to sit or stand so that you can understand your communication partners well, asking others to articulate well or speak more slowly.

   If you are already consciously and actively applying multiple communication strategies, please choose ‘10’.

0	1	2	3	4	5	6	7	8	9	10
Not ready at all										Highly ready

2. How ready are you to **work on your feelings about your diminished hearing***?

   * Think of learning to cope with the challenges or problems around your diminished hearing (learning to accept, to decrease any negative feelings).

   If you are already working on your feelings intensively, please choose '10'.

0	1	2	3	4	5	6	7	8	9	10
Not ready at all										Highly ready

3. How ready are you to **involve others in your diminished hearing***?
   * Think of sharing any challenges and problems around your hearing with others. This can, but doesn't necessarily need to be about sharing feelings.
   If you are already intensively involving others, please choose '10'.

0	1	2	3	4	5	6	7	8	9	10
Not ready at all										Highly ready

4. How ready are you to **try hearing aids**?
   If you have already scheduled or had a hearing aid trial, or already purchased them, please choose ‘10’.

0	1	2	3	4	5	6	7	8	9	10
Not ready at all										Highly ready

5. How ready are you to **try assistive listening devices**?
   For example, think of amplified headphones for the TV, a doorbell amplifier, or a vibrating alarm clock.
   If you have already scheduled or had a trial period for an assistive listening device, or already purchased one, please choose ‘10’.

0	1	2	3	4	5	6	7	8	9	10
Not ready at all										Highly ready

Fig. 1. *Screenshots of the App's Main Pages. The app was offered in Dutch only, but the screenshots were translated to English for this publication.*

Pane 1 - Home screen: Help and navigation to entering new listening situations (via the plus—icon) or viewing entered situations (total and per day), and their snippets. The pie-chart showed the total number of listening situations specified by type.

Pane 2 – EMA survey: Example of a question of the evaluation survey. The 'i'-icon held a short informative text explaining why it generally is more difficult to understand someone in certain types of background noise (e.g., background speech vs. steady noise from an electronic device).

Pane 3 - My listening situations: Help for this view and overview of all entered listening situations, organized by date. New listening situations can be entered here as well (via the plus-icon).

Pane 4 - My most difficult listening situations: A ranked list of the listening situations that were rated on level of difficulty by the user.

Pane 5 - My answers to the short surveys: Per selected listening situation (or for all types of listening situations lumped together), the user's individual and the whole user group's answers to a particular survey question are shown.

Pane 6 - My library: Per theme of the app (e.g., Communication strategies), the user can view, review, and share the snippets available to them.

Pane 7 - Snippet: Example of a snippet, on the value of speech reading. Its main target behavior was *Communication Strategies*, and main Stage of Change was *Preparation* (for the analysis of RQ 3).

Pane 8 – Snippets review screen: When closing a snippet, users were asked to review the snippet on six indicators.

HEAR-AWARE APP: ACCEPTABILITY AND STAGES OF CHANGE

1

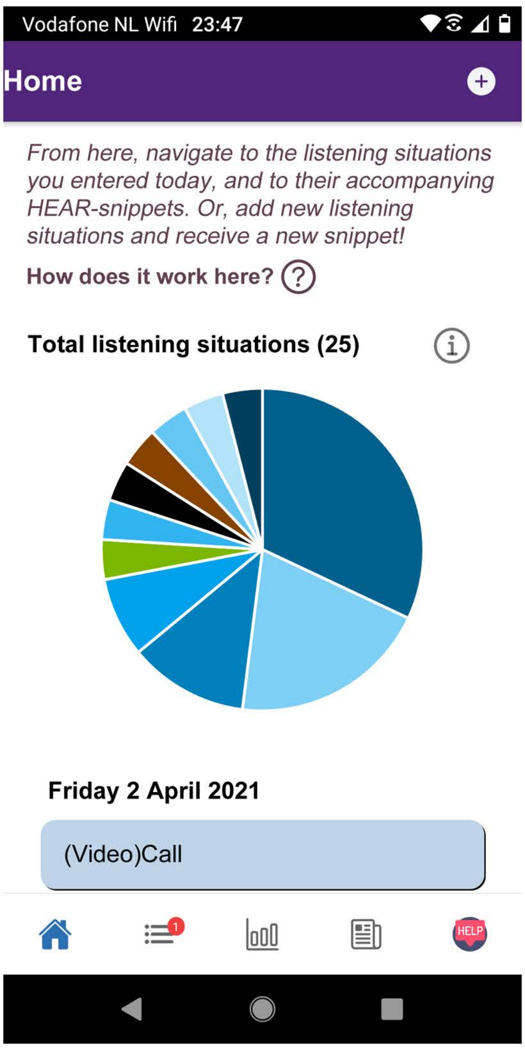

2

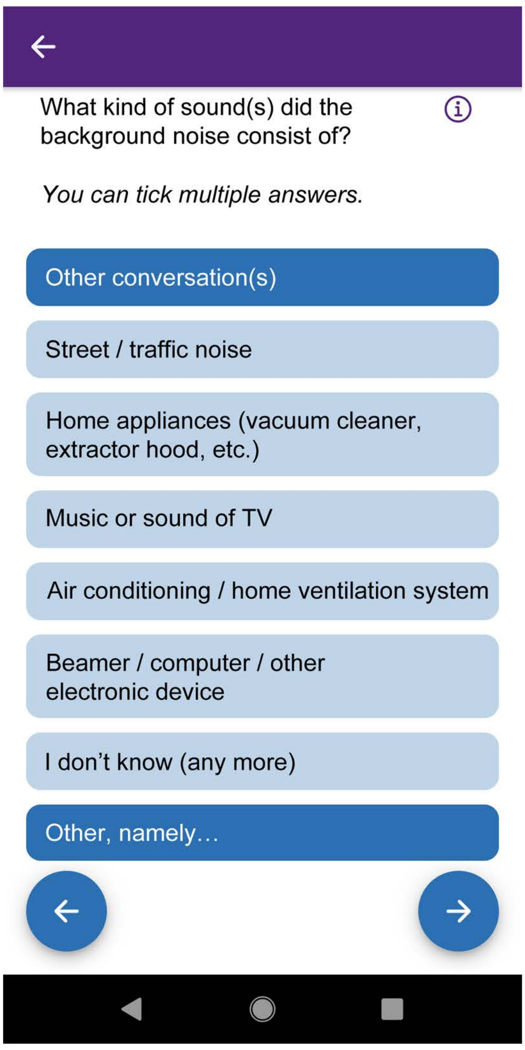

3

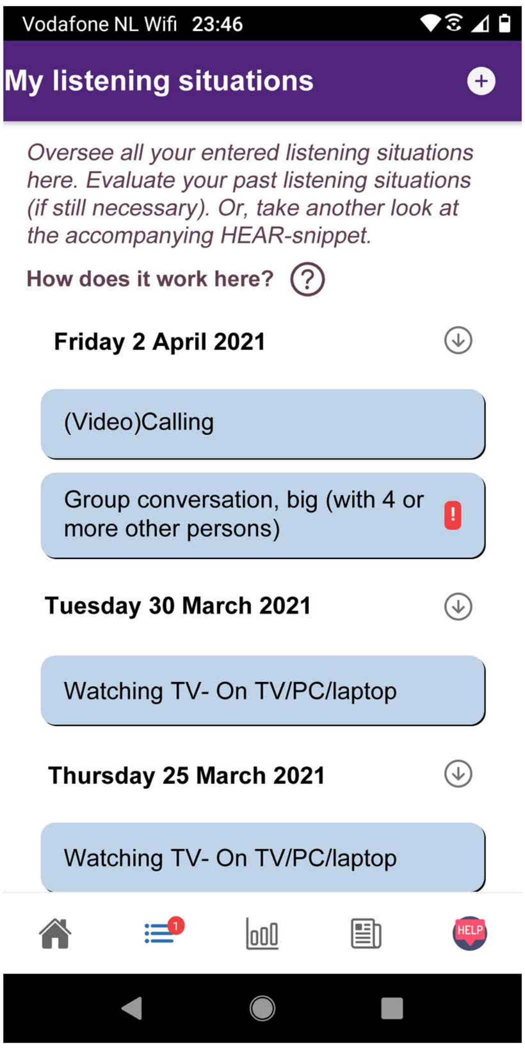

4

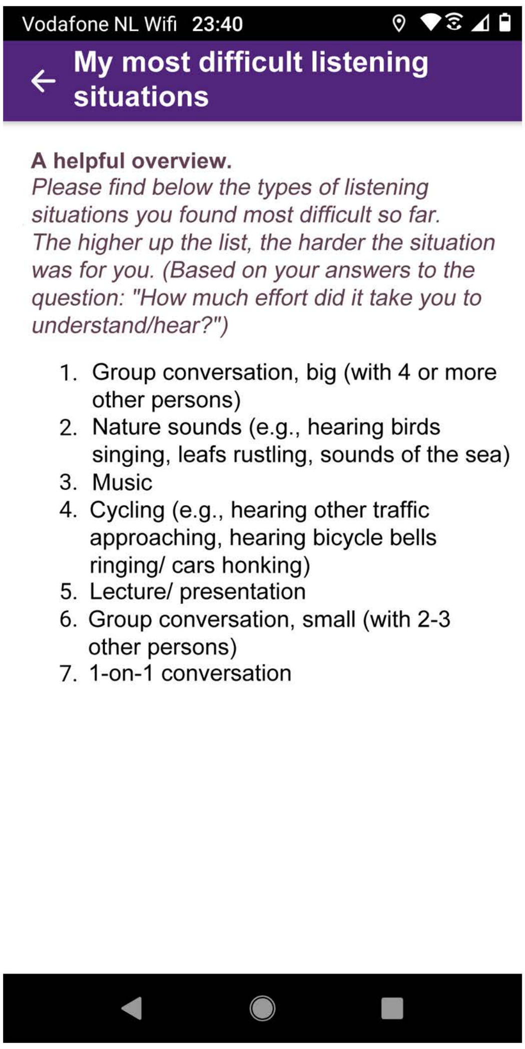

HEAR-AWARE APP: ACCEPTABILITY AND STAGES OF CHANGE

5

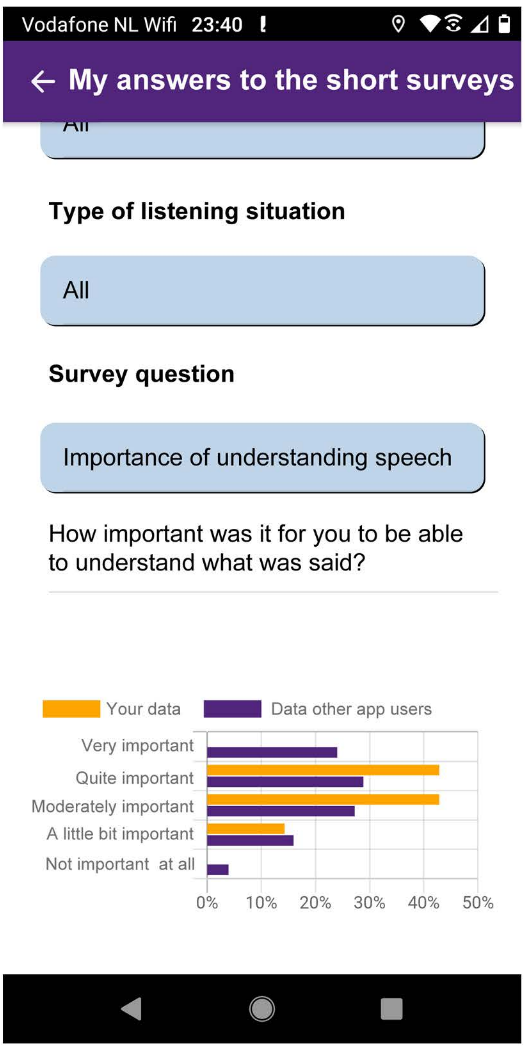

6

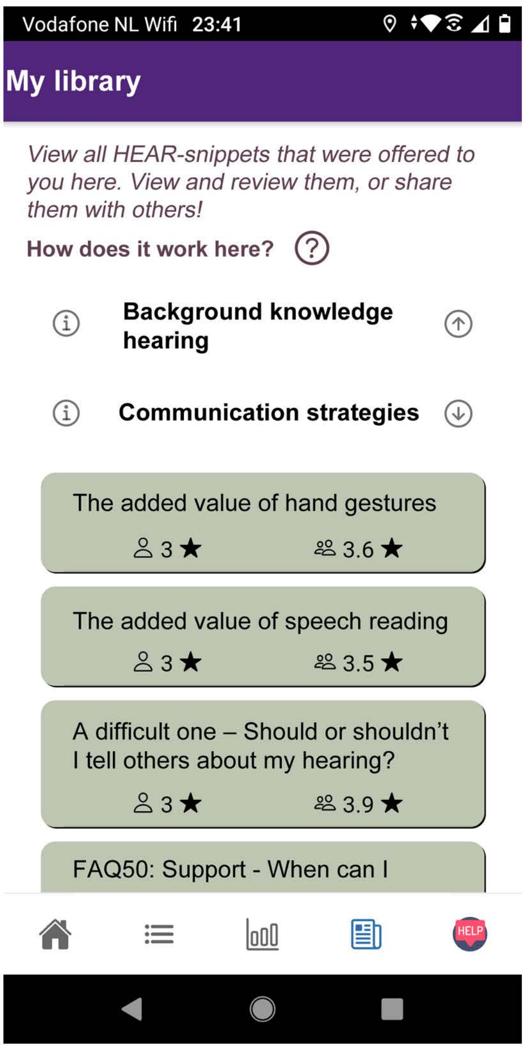

7

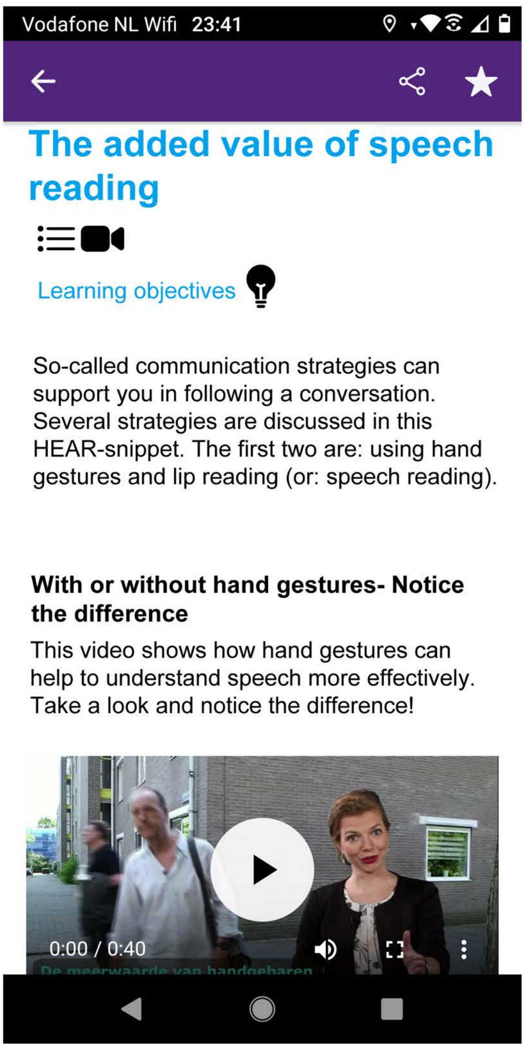

8

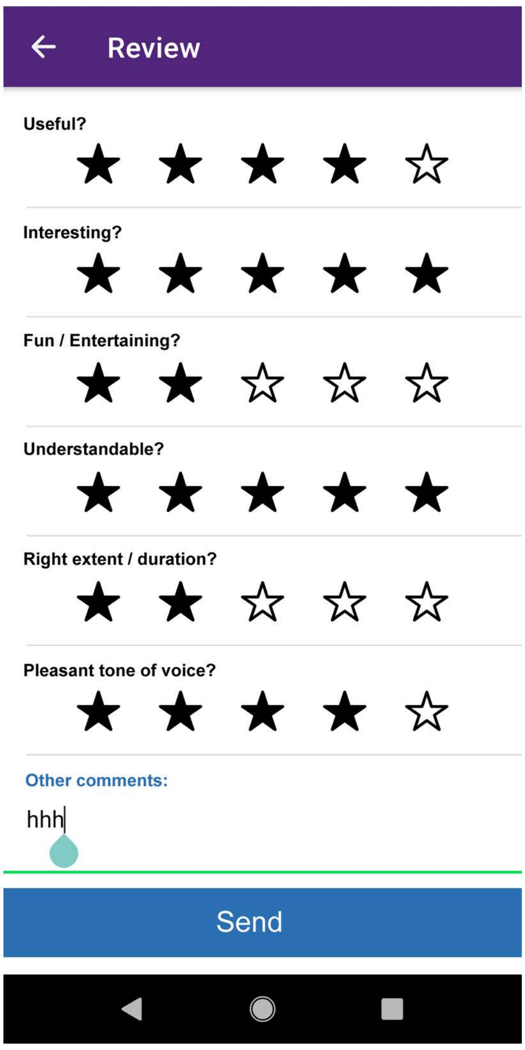

Supplement: Supplementary file 1 [file aud-45-186-s001.pdf]
