# Supplementary material for: Feasibility of the HEAR-aware App for Hearing Loss Self-Management: A Nonrandomized Intervention Study to Examine Intervention Acceptability and the Stages-of-Change Concept
Source: Ear Hear. 2023 Sep 6;45(1):186–97. doi: 10.1097/AUD.0000000000001414 (PMC10718215; doi:10.1097/AUD.0000000000001414)
Supplement: Supplementary file 2 [file aud-45-186-s002.pdf]

Supplemental Digital Content 2: Results

Content:

- **TABLE SR1.** *Reported Reasons of Study Participants for Not Opting for a Hearing Aid (N = 29). Eleven Participants (38%) Reported Multiple Reasons*
- **TABLE SR2.** *Descriptive Sample Statistics (N=26)*
- **TABLE SR3.** *Types of Added Listening Situations by Participants (N =26)*
- **TABLE SR4.** *Descriptive Statistics of the Snippet Review Scores per Indicator, and of the EMA Evaluation Survey Question on the Snippet's Usefulness*
- **TABLE SR5.** *Cross-tabulation of the Staging Algorithm Generic on T0 and T1. ICC = .74 (Good)*
- **TABLE SR6.** *Cross-tabulation of the Staging Algorithm Communication Strategies on T0 and T1. ICC = .72 (Good)*
- **TABLE SR7.** *Cross-tabulation of the Staging Algorithm Hearing Aids on T0 and T1. ICC = .80 (Excellent)*
- **TABLE SR8.** *Cross-tabulation of the Staging Algorithm Emotional Coping on T0 and T1. ICC = .51 (Fair)*
- **TABLE SR9.** *Cross-tabulation of the Staging Algorithm Social Support on T0 and T1. ICC = .72 (Good)*
- **TABLE SR10.** *Cross-tabulation of the Staging Algorithm Assistive Listening Devices on T0 and T1. ICC = .69 (Good)*
- **TABLE SR11.** *Correlation Coefficients (and their 95% Confidence Intervals) of the TB-specific and Generic SoC-scales, and Self-reported Hearing Disability, at T0 (N = 29)*
- **TABLE SR12.** *Frequencies of Combinations of SoC_{person} and $SoC_{snippet}$, with Associated $SoC_{difference}$ Scores Presented between Brackets (N = 25)*

TABLE SR1. *Reported Reasons of Study Participants for Not Opting for a Hearing Aid (N = 29).*

Eleven Participants (38%) Reported Multiple Reasons

	Reason	Frequency
1.	Does not feel ready yet for a HA (not specified)	11
2.	Does not think hearing problems are severe enough	10
3.	Hearing aid retailer or ENT doctor had indicated that hearing loss was 'not that severe yet'	8
4.	HAs too expensive	5
5.	Vanity (visibility HAs)	5
6.	Old-age stigma connected to HAs	2
7.	Low expectations of gain/sound quality	1
8.	Too many other health problems	1
9.	Reason unknown/unclear	2

HA, hearing aid. ENT, Ear Nose, and Throat.

TABLE SR2. *Descriptive Sample Statistics (N = 26)*

Measure	*M* (*SD*) or *Mdn* [25th and 75th Percentile Points] or *N* (%)	Actual Range
Age (years)	63.4 (8.3)	51 - 79
Men	18 (69%)	-
Level of education*		-
Low	2 (8%)	
Medium	18 (69%)	
High	6 (23%)	
Work status (paid work ≥ 1 hrs per wk)		-
Yes	15 (58%)	
No	11 (42%)	
Marital status		-
Married	47 (65%)	
Cohabiting	3 (12%)	
Widowed	1 (4%)	
Divorced	2 (8%)	
Single, never married	3 (12%)	
Hearing loss better ear (PTA, dB HL)	34.2 (7.6)	13.3 - 46.7
Hearing loss poorer ear (PTA, dB HL)	38.3 [35.0 – 47.1]	35.0 - 71.7

dB HL, decibels hearing level; *M*, mean; *Mdn*, median; PTA, pure tone average hearing loss, measured across 1, 2 and 4 kHz; *SD*, standard deviation; -, not applicable.
*Low: uncompleted elementary, elementary, lower vocational; medium: general intermediate, intermediate vocational, general secondary; high: higher vocational, college, and university.

TABLE SR3. *Types of Added Listening Situations by Participants (N = 26)*

Type of Listening Situation	Number of Listening Situations Added (Frequency)	Percentage of Total (Top-5 Indicated in Bold)	Unique Participants* (N)
1. **(Video)calling**	58	**9%**	19
2. **1-on-1 conversation**	115	**19%**	23
3. **Group conversation, small (with 2-3 other persons)**	86	**14%**	22
4. **Group conversation, big (with 4 or more other persons)**	65	**11%**	20
5. Lecture/ presentation	16	3%	10
6. Sports instruction	17	3%	8
7. **Watching TV – On TV/PC/laptop**	88	**14%**	20
8. Watching clips – On smartphone	6	1%	5
9. Radio interview / podcast / audiobook	7	1%	5
10. Music	12	2%	6
11. Cycling *(e.g., hearing other traffic approaching, hearing bicycle bells ringing/ cars honking)*	33	5%	11
12. Driving the car *(e.g., hearing other cars honking, emergency vehicles sirens)*	23	4%	12
13. Using public transport *(e.g., hearing check-in/check-out signals, public address systems)*	7	1%	4
14. Walking *(e.g., hearing other traffic approaching, hearing bicycle bells ringing/ cars honking)*	13	2%	6
15. Nature sounds *(e.g., hearing birds singing, leafs rustling, sounds of the sea)*	13	2%	6
16. Other, namely…	57	9%	14
Total	616	100%	-

* Unique number of participants that added ≥1 listening situations of the particular type.

TABLE SR4. *Descriptive Statistics of the Snippet Review Scores per Indicator, and of the EMA Evaluation Survey Question on the Snippet's Usefulness. Presented for All Snippets Lumped Together (Top Row), and Different Snippet Subgroups (Lower Rows). Unless Indicated Otherwise, Medians and 25th and 75th Percentile Points Are Presented.*

	Snippet Review Scores (Possible Range 1-5)							EMA Evaluation Survey Question on Snippet Usefulness*		
	No. of Observations (Unique SNs; Participants)	Useful	Interes-ting	Fun/ Enter-taining	Understan-dable	Appealing Tone	Appropriate length/ duration	No. of Observations (Unique SNs; Participants)	Response Options	No. of Obser-vations (%)
All SNs	635 (107; n=25)	4.0 [3.0; 5.0]	4.0 [3.0; 5.0]	3.0 [2.0; 4.0]	4.0 [4.0; 5.0]	4.0 [3.0; 4.0]	4.0 [3.0; 4.0]	-	-	-
Offered SNs	424 (70; n=24)	4.0 [3.0; 5.0]	4.0 [3.0; 5.0]	3.0 [2.0; 4.0]	4.0 [4.0; 5.0]	4.0 [3.0; 4.0]	4.0 [3.0; 4.0]	313 (70; n=23)	1) No<br>2) A bit<br>3) Much<br>4) Very much	110 (35%)<br>158 (50%)<br>40 (13%)<br>5 (2%)
Offered SNs- acoustically tailored◊	322 (46; n=23)	4.0 [3.0; 5.0]	4.0 [3.0; 5.0]	3.0 [2.0; 4.0]	4.0 [4.0; 5.0]	4.0 [3.0; 4.0]	4.0 [3.0; 5.0]	228 (45; n=22)	1) No<br>2) A bit<br>3) Much<br>4) Very much	67 (29%)<br>124 (54%)<br>33 (15%)<br>4 (2%)
Offered SNs- not acoustically tailored◊	102 (33; n=19)	4.0 [3.0; 5.0]	4.0 [3.0; 5.0]	2.0 [1.0; 3.0]	4.0 [4.0; 5.0]	3.0 [3.0; 4.0]	4.0 [3.0; 4.0]	85 (33; n=17)	1) No<br>2) A bit<br>3) Much<br>4) Very much	43 (51%)<br>34 (40%)<br>7 (8%)<br>1 (1%)

Standard library SNs	211 (37; n=22)	4.0 [3.0; 5.0]	4.0 [4.0; 5.0]	3.0 [2.0; 4.0]	4.0 [4.0; 5.0]	4.0 [3.0; 4.0]	4.0 [3.0; 4.0]	-	-	-
All SNs with main TB Communication Strategies	115 (9; n=24)	4.0 [3.0; 4.0]	4.0 [3.0; 4.0]	3.0 [2.0; 4.0]	4.0 [4.0; 5.0]	4.0 [3.0; 4.0]	4.0 [3.0; 4.0]	68 (8; n=21)	1) No<br>2) A bit<br>3) Much<br>4) Very much	13 (19%)<br>39 (57%)<br>15 (22%)<br>1 (2%)
All SNs with main TB Hearing Aids	111 (23; n=22)	4.0 [3.0; 5.0]	4.0 [3.0; 4.0]	3.0 [1.0; 4.0]	4.0 [4.0; 5.0]	4.0 [3.0; 4.0]	4.0 [3.0; 4.0]	53 (16; n=16)	1) No<br>2) A bit<br>3) Much<br>4) Very much	23 (43%)<br>24 (45%)<br>5 (10%)<br>1 (2%)
All SNs with main TB Emotional Coping	28 (5; n=14)	4.0 [4.0; 5.0]	4.0 [4.0; 5.0]	3.0 [2.0; 4.0]	4.0 [4.0; 5.0]	4.0 [3.0; 4.0]	4.0 [4.0; 4.0]	22 (4; n=12)	1) No<br>2) A bit<br>3) Much<br>4) Very much	6 (27%)<br>14 (64%)<br>2 (9%)<br>0 (0%)
All SNs with main TB Social Support	34 (3; n=16)	4.0 [3.8; 5.0]	4.0 [3.8; 5.0]	3.0 [2.0; 4.3]	4.0 [4.0; 5.0]	4.0 [3.0; 5.0]	4.0 [4.0; 5.0]	27 (3; n=16)	1) No<br>2) A bit<br>3) Much<br>4) Very much	7 (26%)<br>19 (71%)<br>1 (4%)<br>0 (0%)
All SNs with main TB ALDs without hearing aids	20 (3; n=14)	3.5 [3.0; 4.8]	3.5 [3.0; 4.0]	3.0 [1.3; 4.0]	4.0 [4.0; 5.0]	4.0 [3.3; 4.0]	4.0 [3.0; 4.8]	10 (2; n=9)	1) No<br>2) A bit<br>3) Much<br>4) Very much	5 (50%)<br>3 (30%)<br>2 (20%)<br>0 (0%)

All SNs with main TB ALDs with hearing aids	39 (4; n=18)	4.0 [3.0; 5.0]	4.0 [3.0; 5.0]	3.0 [2.0; 4.0]	4.0 [4.0; 5.0]	4.0 [3.0; 4.0]	4.0 [3.0; 4.0]	32 (4; n=16)	1) No<br>2) A bit<br>3) Much<br>4) Very much	11 (34%)<br>15 (47%)<br>4 (13%)<br>2 (6%)
All SNs with no TB	288 (60; n=24)	4.0 [3.0; 5.0]	4.0 [4.0; 5.0]	3.0 [2.0; 4.0]	4.0 [4.0; 5.0]	4.0 [3.0; 4.0]	4.0 [3.0; 4.0]	115 (37; n=21)	1) No<br>2) A bit<br>3) Much<br>4) Very much	46 (40%)<br>55 (48%)<br>13 (11%)<br>1 (1%)

ALDs, Assistive listening devices; EMA, Ecological Momentary Assessment, No., number; SN, snippet; TB, target behavior; -, no data, not applicable.

* Did the information / tips from the snippet that you received earlier today for this listening situation help you in any way?

◊ Acoustically tailored snippets: snippets that had 1-3 matching labels with the 1-3 acoustic labels activated during the EMA identification survey. Non-acoustically tailored snippets: snippets that had no acoustic label activated.

Tables SR5-SR10 show the cross-tabulations of the test-retest reliability results for the different Staging Algorithm measures (*Intraclass Correlation Coefficients*, *ICCs*, single measurement, absolute agreement, 2 way mixed effects model).

TABLE SR5. *Cross-tabulation of the Staging Algorithm Generic on T0 and T1. ICC = .74 (Good)*

	**SoC on T1**				
**SoC on T0**	Precontemplation	Contemplation	Preparation	Action	Total
Precontemplation	0	0	0	0	0
Contemplation	0	12	2	0	14
Preparation	0	4	6	1	11
Action	0	0	1	3	4
Total	0	16	9	4	29

SoC, Stage of Change.

TABLE SR6. *Cross-tabulation of the Staging Algorithm Communication Strategies on T0 and T1. ICC = .72 (Good)*

	**SoC on T1**				
**SoC on T0**	Precontemplation	Contemplation	Preparation	Action	Total
Precontemplation	3	2	0	0	5
Contemplation	2	7	1	1	11
Preparation	0	1	3	4	8
Action	0	1	0	4	5
Total	5	11	4	9	29

SoC, Stage of Change.

TABLE SR7. *Cross-tabulation of the Staging Algorithm Hearing Aids on T0 and T1. ICC = .80 (Excellent)*

	SoC on T1				
**SoC on T0**	Precontemplation	Contemplation	Preparation	Action	Total
Precontemplation	1	0	0	0	1
Contemplation	2	20	1	0	23
Preparation	0	0	5	0	5
Action	0	0	0	0	0
Total	3	20	6	0	29

SoC, Stage of Change.

TABLE SR8. *Cross-tabulation of the Staging Algorithm Emotional Coping on T0 and T1. ICC = .51 (Fair)*

	SoC on T1				
**SoC on T0**	Precontemplation	Contemplation	Preparation	Action	Total
Precontemplation	13	0	2	1	16
Contemplation	0	2	1	1	4
Preparation	0	1	2	3	6
Action	1	1	0	1	3
Total	14	4	5	6	29

SoC, Stage of Change.

TABLE SR9. *Cross-tabulation of the Staging Algorithm Social Support on T0 and T1. ICC = .72 (Good)*

	SoC on T1				
**SoC on T0**	Precontemplation	Contemplation	Preparation	Action	Total
Precontemplation	6	1	1	0	8
Contemplation	2	4	1	0	7
Preparation	1	0	3	3	7
Action	1	0	0	6	7
Total	10	5	5	9	29

SoC, Stage of Change.

TABLE SR10. *Cross-tabulation of the Staging Algorithm Assistive Listening Devices on T0 and T1. ICC = .69 (Good)*

	SoC on T1				
**SoC on T0**	Precontemplation	Contemplation	Preparation	Action	Total
Precontemplation	6	0	0	0	6
Contemplation	4	14	2	0	20
Preparation	0	1	1	0	2
Action	0	0	1	0	1
Total	10	15	4	0	29

SoC, Stage of Change.

TABLE SR11. *Correlation Coefficients (and their 95% Confidence Intervals) of the TB-specific and Generic SoC-scales, and Self-reported Hearing Disability, at T0 (N = 29)*

The Line (0 - 10)	Generic	Communication Strategies	Hearing Aids	Emotional Coping	Social Support	ALDs	Self-reported Hearing Disability (0 - 74)
Generic	1	**.47*** (.10 - .72)	**.44*** (.09 - .70)	.36 (-.01 - .64)	.08 (-.29 - .44)	.15 (-.23 - .49)	.17 (-.21 - .50)
Communication Strategies	**.47*** (.10 - .72)	1	.10 (-.28 - .45)	.36 (-.02 - .65)	**.74**** (.48 - .88)	.19 (-.19 - .52)	-.16 (-.50 - .22)
Hearing Aids	**.44*** (.09 - .70)	.10 (-.28 - .45)	1	.17 (-.21 - .51)	-.17 (-.50 - .21)	.22 (-.16 - .54)	**.39*** (.03 - .66)
Emotional Coping	.36 (-.01 - .64)	.36 (-.02 - .65)	.17 (-.21 - .51)	1	.19 (-.19 - .52)	-.01 (-.38 - .35)	-.24 (-.56 - .14)
Social Support	.08 (-.29 - .44)	**.74**** (.48 - .89)	-.17 (-.50 - .21)	.19 (-.19 - .52)	1	.10 (-.28 - .45)	.02 (-.35 - .39)
ALDs	.15 (-.23 - .49)	.190 (-.19 - .52)	.22 (-.16 - .54)	-.01 (-.38 - .35)	.10 (-.28 - .45)	1	.23 (-.15 - .55)
**Staging Algorithm (0 - 3)**	**Generic**	**Communication Strategies**	**Hearing Aids**	**Emotional Coping**	**Social Support**	**ALDs**	**Self-reported Hearing Disability (0 - 74)**
Generic	1	.21 (-.12 - .53)	**.25* (-.07 - .54)**	.11 (-.15 - .40)	.06 (-.31 - .41)	.11 (-.12 - .39)	.15 (-.24 – 0.50)
Communication Strategies	.21 (-.12 - .53)	1	-.05 (-.38 - .31)	**.34* (.01 - .62)**	**.70** (.45-.85)**	.22 (-.10 - .51)	-.20 (-.54 – .19)
Hearing Aids	**.25 * (-.07 - .54)**	-.05 (-.38 - .31)	1	.08 (-.28 - .42)	-.08 (-.42 - .28)	.14 (-.21 - .47)	**.45*** (.09 – .71)
Emotional Coping	.11 (-.15 - .40)	**.34* (.01 - .62)**	.08 (-.28 - .42)	1	**.55* (.19 - .77)**	**.52* (.20 - .75)**	-.22 (-.55 – .17)

Social Support	.06<br>(-.31 - .41)	**.70****<br>**(.45 - .85)**	-.08<br>(-.42 - .28)	**.55***<br>**(.19 - .77)**	1	.14<br>(-.21 - .47)	-.23<br>(-.56 – .16)
ALDs	.11<br>(-.12 - .39)	.22<br>(-.10 - .51)	.14<br>(-.21 - .47)	**.52***<br>**(.20 - .75)**	.14<br>(-.21 - .47)	1	-.09<br>(-.45 - .30)

* Statistically significant ($p < .05$), but correlation coefficient < .60;

** Statistically significant ($p < .01$), and correlation coefficient ≥ .60

ALDs, Assistive Listening Devices without hearing aids.

TABLE SR12. *Frequencies of Combinations of SoC_{person} and $SoC_{snippet}$, with Associated $SoC_{difference}$ Scores Presented between Brackets (N = 25)*

**$SoC_{snippet}$**	**SoC_{person}**				
	Precontemplation	Contemplation	Preparation	Action	Total
Precontemplation	3 (0)	78 (-1)	12 (-2)	19 (-3)	112
Contemplation	17 (1)	222 (0)	64 (-1)	57 (-2)	360
Preparation	26 (2)	68 (1)	35 (0)	34 (-1)	163
Action	0	0	0	0	0
Total	46	368	111	110	635

Note. One participant had not reviewed any snippets, so data of 25 participants were used in the analyses. SoC, Stage of Change.
